# First-Principles Kinetic Monte Carlo Simulations for Single-Cluster Catalysis: Study of CO_2_ and CH_4_ Conversion on Pt/HfC

**DOI:** 10.1021/acscatal.4c07877

**Published:** 2025-02-03

**Authors:** Hector Prats, Michail Stamatakis

**Affiliations:** † Department of Chemistry, Physical & Theoretical Chemistry Laboratory, University of Oxford, South Parks Road, Oxford OX1 3QZ, U.K.; ‡ Institute of Materials Chemistry, Technische Universität Wien, 1060 Vienna, Austria; § Department of Chemistry, Inorganic Chemistry Lab, University of Oxford, Oxford OX1 3QR, U.K.

**Keywords:** Kinetic Monte Carlo, single-cluster catalyst, transition metal carbides, kinetic modeling, CO_2_ conversion, methane reforming, density functional theory, dry-reforming

## Abstract

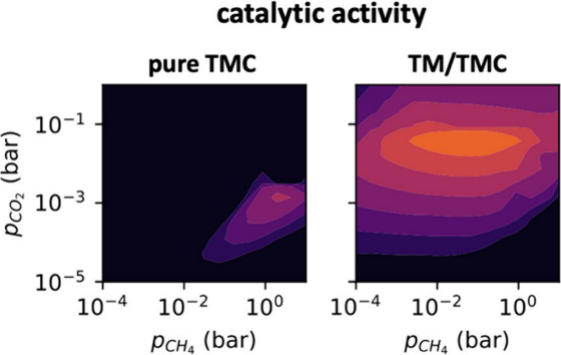

The deposition of small transition metal (TM) clusters on transition metal carbides (TMC) gives rise to bifunctional catalysts with multiple active sites. This family of single-cluster catalysts (SCCs) offers exciting opportunities for enabling a wider range of chemical reactions owing to their strong metal–support interactions, which drastically modify the catalytic properties of the supported metal atoms. In this work, we use first-principles Kinetic Monte Carlo (KMC) simulations to investigate the conversion of CO_2_ and CH_4_ on Pt/HfC, which was identified as the most promising TM/TMC combination in a previous DFT-based high-throughput screening study. We analyze the interplay between the Pt clusters and the HfC support, evaluating the catalytic activity, selectivity, and adlayer composition across a broad range of operating conditions (*p*_*A*_, *p*_*B*_, and *T*) and Pt loadings. This study evaluates five different industrial processes, including the dry reforming (DRM), steam reforming (SRM), and partial oxidation (POM) of methane, as well as the water–gas shift (WGS) reaction and its reverse (RWGS). Our results demonstrate that the deposition of Pt clusters on HfC systematically enhances the catalytic performance, even at a Pt loading as low as ∼0.02 ML. This work illustrates the extensive catalytic benefits of SCCs and highlights the importance of considering diffusion steps and lateral interactions in kinetic modeling.

## 1 Introduction

Advancing the field of heterogeneous catalysis relies on the rational design of novel materials with a targeted functionality, which ultimately requires an atomistic-scale understanding of the interplay between the different active sites and a large number of elementary steps. Despite the useful theoretical insights into the molecular reaction mechanism that can be obtained by analyzing the potential or Gibbs free energy profiles typically obtained from density functional theory (DFT) calculations, this information is in general insufficient to make sound predictions of catalytic activity. This is, in part, because most relevant catalytic systems involve many elementary steps and/or complex catalysts exhibiting different active regions.^1,2^ Moreover, this analysis does not account for coverage effects such as lateral-interactions-dependent reaction rates or site blocking, which can play a key role. For instance, a recent study on the reverse water–gas shift (RWGS) reaction on Ni/TiC showed how the predictions from free energy profiles can be in complete disagreement with those of kinetic simulations as a consequence of strong coverage effects.^3^ While coverage effects can be captured to some extent by prevalent microkinetic modeling approaches through coverage-dependent rate expressions, the mean-field approximation can fail to capture correlations, fluctuations, and special distributions of adsorbed species, which are crucial to determine the catalytic activity and selectivity, even for simple catalysts such as Rh.^4,5^ Kinetic Monte Carlo (KMC) simulations, on the other hand, naturally capture coverage effects beyond the mean-field approximation,^6,7^ and can be used to understand and potentially rationally design novel catalysts.

In this paper, we use KMC simulations to study the time-evolution at the catalyst surface of a system consisting of small Pt clusters supported on HfC, denoted as Pt/HfC. In a recent DFT-based high-throughput screening study,^8^ Pt/HfC was identified as the most promising catalyst among all combinations between 7 transition metal (TM) clusters supported on 11 stable surfaces of transition metal carbides (TMCs) toward the conversion of CO_2_ and CH_4_, due to its predicted good stability and ability to dissociate both CO_2_ and CH_4_ with a very low energy barrier. Earth-abundant TMCs have gained popularity in the last decades because they are highly refractory and resist sintering, coking, and sulfur poisoning.^9^ Importantly, TMC catalysts show properties similar to those of noble metals^10^ but are much cheaper. Apart from their catalytic activity *per se*, TMCs are excellent substrates to disperse small metal clusters due to the strong covalent interactions which immobilize the supported clusters,^11,12^ but also due to the significant polarization of the electron density of the metal clusters induced by the TMC which boosts their catalytic activity in a way that it can be much superior to that of clusters dispersed on more traditional oxide supports.^13,14^ For instance, small Ni clusters supported on TiC have been shown to activate CH_4_ at room temperature,^15^ and Au, Cu and Ni clusters are more active when supported on TiC compared to traditional oxide supports for CO_2_ hydrogenation.^16^ Therefore, supported TM particles on TMCs (i.e., TM/TMC) comprise a subtype of a broader family of materials consisting of small, supported clusters, referred to as single-cluster catalysts (SCCs),^17,18^ and offer many exciting opportunities for designing novel catalysts with tailored performance in various catalytic applications.

One of the most promising applications for TM/TMC is the conversion of CH_4_ to value-added fuels and chemicals. This process is typically achieved via the formation of syngas, a mixture of CO and H_2_ that can be later transformed to e.g. hydrocarbons via Fischer–Tropsch synthesis,^19^ methanol,^20^ or dimethyl ether.^21^ Production of syngas out of methane can be done using dry reforming (DRM), steam reforming (SRM) and partial oxidation (POM) reactions (eqs 1-3), typically coupled with the water–gas shift (WGS, eq 4) or its reverse (RWGS) to balance the H_2_/CO ratio:
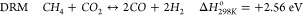
1
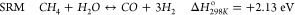
2
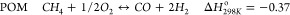
3
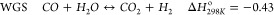
4

Among these three processes, DRM represents a very interesting approach thanks to the simultaneous conversion of both CO_2_ and CH_4_ to more valuable chemicals and/or fuels, as well as to the prospect of reducing the overall carbon footprint in the atmosphere.^22,23^ However, DRM is still in its nascent stage.^24^ The major hurdle preventing the industry-wide application of DRM is coke formation and sintering, which deactivate catalysts rapidly.^25,26^ Moreover, the thermodynamic barrier of DRM is a big challenge that hinders high conversion levels of the two molecules at mild temperatures (without the use of significant energy input). Strong intramolecular bonds render both molecules chemically inert and thermodynamically stable,^27,28^ therefore requiring the rational design of highly active and stable catalysts. This is where highly active SCCs such as TM/TMCs can play an important role.

Motivated by these challenges, a large part of the present work is devoted to studying the catalytic performance of Pt/HfC for the DRM, SRM, POM, WGS and RWGS reactions on a wide range of operating conditions (*p*_*A*_, *p*_*B*_, and *T*) and Pt loading, and comparing it to that of the bare HfC. By means of KMC simulations, we analyze the interplay between the Pt cluster and the HfC support on the reaction mechanism, identify the operating conditions that maximize activity and/or selectivity, and unveil the origin of the predicted synergistic effects. Moreover, we assess the employed methodology and show that the incorporation of diffusion processes and lateral interactions can play a critical role in the KMC simulation results.

## 2 Methods

### 2.1 DFT Calculations

**Figure 1:**
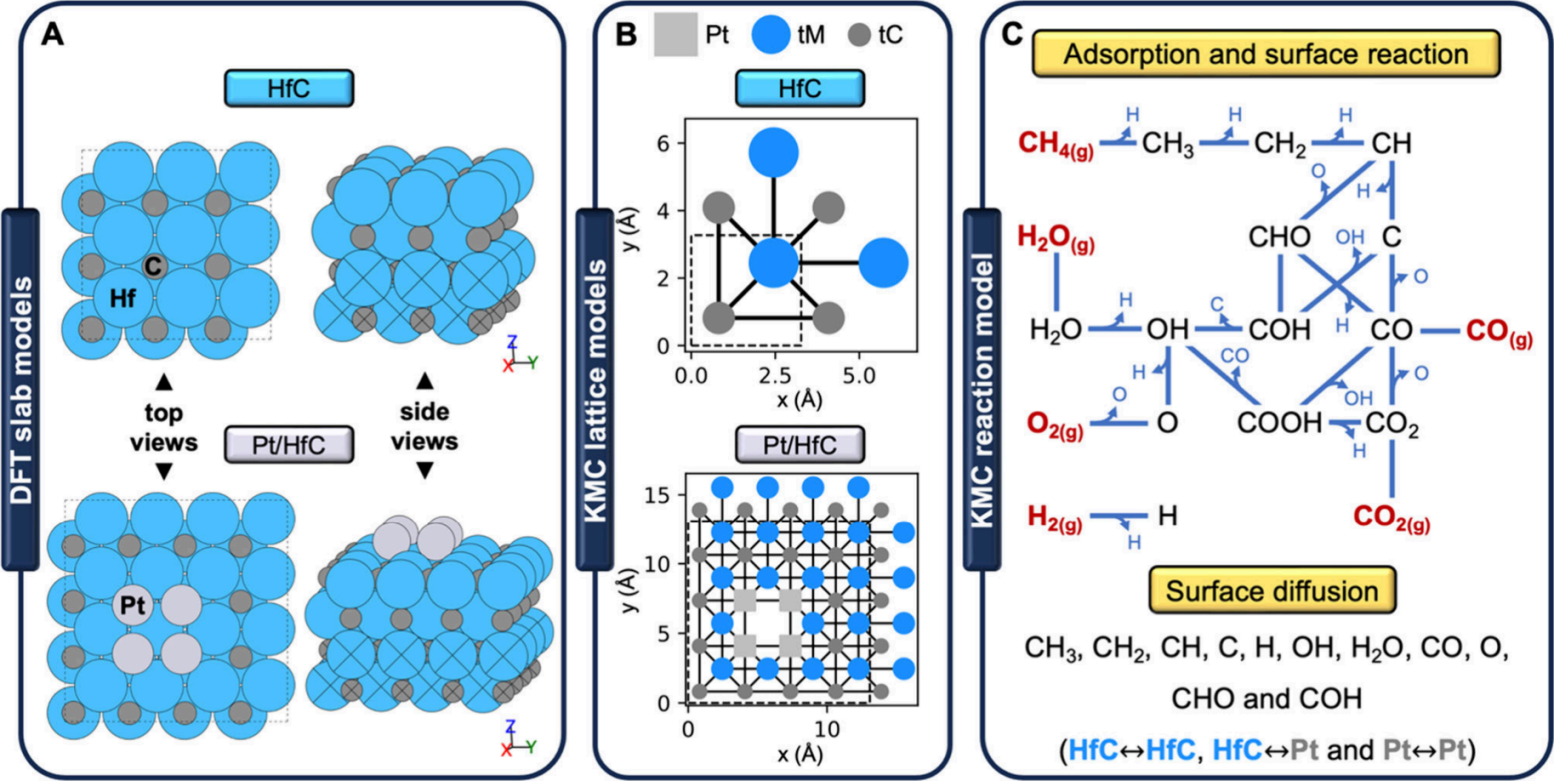
(A) DFT slab models for HfC (top) and Pt/HfC (bottom). (B) KMC lattice models for HfC and Pt/HfC. The unit cells are shown in thick dashed lines, while solid lines indicate site connectivity. (C) KMC reaction model, common for all five reactions considered in this study.

All periodic DFT calculations were carried out using the Vienna *Ab-initio* Simulation Package (VASP), version 5.4.4.^29^ The PBE exchange-correlation functional^30^ was used, which provides the most accurate results among GGA functionals in describing the atomic and electronic structure of TMCs.^31^ Dispersion (van der Waals) interactions were included through the D3 method as proposed by Grimme et al.^32^ Plane-wave kinetic energy cutoffs of 520 and 415 eV were used for bulk and surface calculations, respectively. The core electrons were described by the projector-augmented wave (PAW) method.^33,34^ The bulk structure of HfC was obtained from the Materials Project open data set.^35^ For bulk geometry relaxation, electronic and force convergence tolerances of 10^–6^ eV and 10^–3^ eV·Å^–1^, respectively, were imposed, and a dense Γ-centered k-point grid of 80/*a* × 80/*b* × 80/*c* was used, with noninteger values rounded up to the nearest integer. For HfC, a 4-layer 3 × 3 HfC(001) slab model was constructed from the optimized bulk structure (Figure 1A), and the bottom half of the slab (in the vertical *z*-direction) was constrained at the bulk positions. For Pt/HfC, a 4-layer 4 × 4 HfC(001) slab model was constructed, and a 4-atom Pt cluster was supported on it (Figure 1A). This size features a compact, high-symmetry structure that maximizes the atomic coordination with the support and is thus energetically more stable. Moreover, previous experimental^36−38^ and theoretical^15,39^ studies have shown that the activity is higher when the size of these clusters is very small (<0.6 nm). The most stable configuration of the 4-atom Pt cluster is taken, as determined in our previous study.^11^

For all DFT calculations of adsorbed configurations and transition states, electronic and force convergence tolerances of 10^–5^ eV and 10^–2^ eV·Å^–1^, respectively, were imposed, and a Γ-centered k-point grid of 60/*a* × 60/*b* × 1 was used, with noninteger values rounded up to the nearest integer. Periodic slab images were separated by 18 Å of vacuum, and a dipole correction was applied to all slab calculations. Transition states were located using CatLearn’s Bayesian transition state search module (ML-NEB)^40^ and, if needed, refined with the improved Dimer method.^41^ All crystal structure manipulations and data analysis were carried out using the Python Materials Genomics package (pymatgen)^42^ and the Atomic Simulation Environment (ASE).^43^

### 2.2 KMC Simulations

All kinetic simulations were carried out using the graph-theoretical KMC approach^44^ combined with cluster expansion Hamiltonians^6,45^ for the surface energetics, as implemented in the *Zacros* code.^44^ The pre-exponential factors for each elementary step have been calculated from the ideal gas and harmonic oscillator models for gas-phase and adsorbed species, respectively, following the procedure described in refs (44) and (46).Further details on the calculation of rate-constants in *Zacros* are provided in Note S1 in the Supporting Information (SI).

The preparation, data analysis and plotting of all simulation results have been done with the Python library *ZacrosTools*.^47^ The KMC lattice models are built so as to mimic the slab model for the DFT calculations and consist of a periodic custom grid of points representing surface sites, where the different species can adsorb, desorb, react or diffuse. Lattice top metal −tM− and top carbon −tC− sites represent the surface tM and tC sites, and the Pt cluster is described in the KMC lattice as four Pt sites, one for each Pt atom (Figure 1B). Specifically, 10 × 10 and 3 × 3 tilings of the unit cells shown in Figure 1B are used to construct the lattices of HfC and Pt/HfC respectively, resulting in a total of 200 and 279 lattice sites. Care is taken to obtain results that are converged with respect to the lattice size (Figure S1). KMC results for Pt/HfC correspond to a Pt loading of 0.25 ML (i.e., Pt_0.25 ML_/HfC) unless otherwise indicated. The Pt loading is calculated as the number of Pt atoms divided by the number of surface C atoms.

Note that catalyst surfaces are inherently dynamic, with processes such as cluster fragmentation, sintering, and reshaping potentially creating new active sites during reaction conditions.^48,49^ While incorporating these dynamic changes into kinetic simulations would provide a more comprehensive understanding of catalytic behavior, it is extremely challenging, as it would require the use of dynamic lattice models, which are beyond the scope of our current study. To support our static lattice model choice, we conducted additional DFT calculations to assess the stability of the Pt_4_ cluster in the presence of CO. Specifically, we investigated the fragmentation pathway of a fully CO-covered Pt_4_ cluster into a Pt_3_ cluster and a single Pt atom (Figure S24). Our calculations indicate that this fragmentation is endothermic (+0.50 eV) and has a substantial energy barrier (∼1.5 eV). These findings indicate that, under the reaction conditions considered, the Pt_4_ cluster remains relatively stable, and that fragmentation is not thermodynamically favorable, thereby supporting our use of a static lattice model.

We assume that all adsorbates occupy only the most stable site in the *HfC region* (i.e., the region of the lattice model comprising all tM and tC sites). Specifically, the tM site is preferred for OH and H_2_O, while the tC site is preferred for all other species. This preference can be explained by the partial charge distribution in HfC: Hf atoms are partially positively charged, and C atoms are partially negatively charged. Accordingly, OH and H_2_O (which bind through the O atom carrying a negative partial charge) are electrostatically stabilized by the positively charged Hf sites. Conversely, CO and CO_2_ bind to the surface via their carbon atom, which is positively charged, thus favoring interaction with the negatively charged C sites. For CH_*x*_ species (*x* = 0 to 3), which also bind to the surface via C, the preference for the C-termination is attributed to the formation of strong covalent C–C bonds. These observations are consistent with general trends reported for TMCs in the literature.^50^

On the Pt clusters, some species prefer to adsorb in bridge or hollow positions; for example, COOH adsorbs on a bridge position between two neighboring Pt atoms. This adsorption mode prevents other adsorbates from occupying the neighboring Pt sites. Therefore, we treat all adsorbate species adsorbing on bridge or hollow positions as multidentate species that occupy all neighboring Pt sites. The reaction list in Table S1 shows the number of sites occupied by each adsorbate. Note that C and CH species adsorb on the hollow site of the cluster, which in the KMC model blocks all four Pt sites from adsorbing other species. However, this assumption might be unrealistic for small H_2_ species, which should still be able to interact with and dissociate at the edges of the cluster. We confirmed using DFT calculations that the presence of C or CH species at the center of the cluster does not prevent H_2_ molecules from dissociating at the cluster’s edge (see Figure S25). To address this issue, we included two additional reactions in the model that allow H_2_ to adsorb and dissociate even when C or CH is adsorbed on the cluster.

The reaction network depicted in Figure 1C and summarized in Table S1 is shared by all reactions. For Pt/HfC, it involves a total of 80 reversible elementary steps (i.e., 16 adsorptions, 34 surface reactions, and 30 surface diffusions). The reaction model for HfC is obtained from that of Pt/HfC by eliminating all steps involving Pt sites. The energetics model includes 31 one-body terms corresponding to the formation energies of the gas-phase and adsorbed species on the different sites (Table S2), and 149 two-body terms corresponding to the pairwise lateral interactions between all possible reactant/product pairs and all possible first-nearest-neighbors (1NN) coadsorbed species on the HfC region (Table S3) and the Pt clusters (Table S4). 2NN and longer-range interactions would substantially increase the complexity of the model and are thus omitted in the current work. In *Zacros*, the effect of lateral interactions on the energy barriers is modeled using the Brønsted-Evans–Polanyi relations^45^ (see Note S2 in the SI for more details).

For every reaction (i.e., DRM, SRM, POM, WGS and RWSG) on each catalyst (i.e., HfC and Pt/HfC), the activity toward every possible product −measured as turnover frequency or TOF−, as well as the selectivity and the coverage of adsorbed species on each site type, are sampled over a wide range of partial pressures for reactants A and B that spans over 5 orders of magnitude for each reactant and are presented in contour diagrams. These diagrams are built by sampling the pressure space using a logarithmically spaced grid of 15 × 15 points (i.e., 225 KMC simulations in total for every reaction on each catalyst). Figure S2 shows that this sampling provides sufficient resolution. Due to the high number of KMC simulations required for this study (∼6000), a single simulation has been carried out for each operating condition (*p*_*A*_, *p*_*B*_ and *T*). Running several replicas for each simulation is beyond our available computational resources and time constraints. Nevertheless, to ensure that using one replica does not compromise the reliability of our results, we ran three additional replicas (total of four replicas) for all 225 (*p*_*A*_, *p*_*B*_) conditions for the DRM on both HfC (Figure S3) and Pt/HfC (Figure S4). Our analysis showed that the differences between the averaged results and the single simulations were negligible.

All KMC simulations are run for either 24 h (HfC) or 72 h (Pt/HfC) of real time, or until reaching 50,000 s of simulated time, whichever occurred first. To tackle the time-scale separation problem inherent in KMC, we used an algorithm performing dynamic scaling of kinetic constants that accelerates the simulations on the fly, and we upscaled the energy barriers of all symmetric diffusion steps with barriers below 0.40 to 0.40 eV. We performed tests to ensure that this upscaling does not affect the simulation results, aside from enabling the simulations to achieve steady-state faster. Thanks to the speedup provided by the dynamic scaling algorithm, KMC simulations generally get past the equilibration stage very quickly (see Figure S22). This is especially true in all catalytically relevant conditions of *p*_*A*_ and *p*_*B*_ (i.e., at the top of the volcano plot), where the average surface coverage is near zero, making the initial state of the lattice (i.e., an empty lattice) very similar to the steady-state.

To verify that all KMC simulations reached steady-state, we have used a function in *ZacrosTools* that automatically determines whether a simulation has likely achieved the steady-state (see Note S3 in the SI for further details on this procedure and the parameters used in the dynamic scaling algorithm). Additionally, we have applied a conservative criterion by assuming that the equilibration phase corresponds to the first half of the total simulated time, effectively allowing equilibration periods of 12 h for HfC and 36 h for Pt/HfC simulations. Finally, given the high computational cost of determining proximity factors for each step, we adopted a commonly used simplification by assigning a proximity factor of 0.0 to all reversible, nonactivated adsorption steps and 0.5 to all other steps. A detailed justification of this approach is provided in Note S4 of the SI.

## 3 Results and Discussion

### 3.1 Predictions from the Energy Barriers

**Figure 2:**
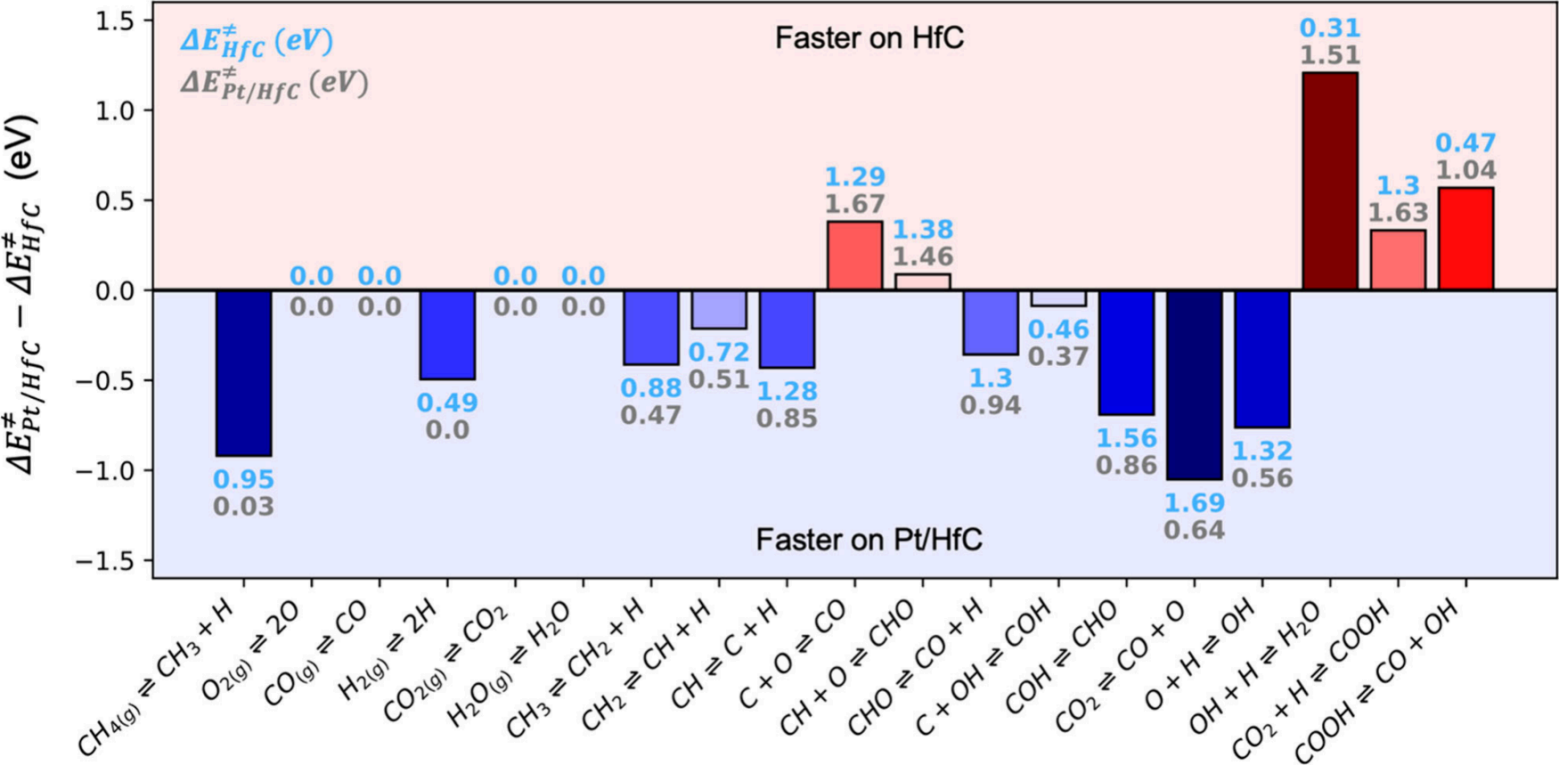
Difference in energy barriers between Pt/HfC Δ*E*_*Pt/HfC*_^≠^) and HfC (Δ*E*_*HfC*_^≠^). Values above or below the bars indicate the energy barriers on HfC (cyan) and Pt/HfC (silver). The color of each bar is indicative of the barrier difference (*y*-axis value). Positive (negative) differences are red (blue), and the magnitude is conveyed by the intensity of the color. All values are in eV, do not include the zero-point energy term, and in the case of two reactants the initial state the barriers are calculated at infinite separation (i.e., not including the lateral interaction). Note that, on Pt/HfC, the elementary steps can occur either on the Pt cluster or at the interface. In this table, only the lowest energy barrier among these two is considered.

We begin this section by comparing the calculated energy barriers on HfC and Pt/HfC and predicting what could be the dominant reaction pathway according to the DFT results alone. Notably, most steps have a lower energy barrier on the very reactive Pt cluster (Figure 2), with the most significant differences being in the dissociative adsorption of CH_4_ (from 0.95 eV on HfC to only 0.03 eV on Pt/HfC) and the dissociation of adsorbed CO_2_ (from 1.69 eV on HfC to only 0.64 eV on Pt/HfC). These are two key steps for DRM, since they are predicted to be rate-limiting due to the high thermodynamic stability of both molecules (i.e., bond strengths of 5.5 and 4.5 eV for the C–O and C–H bonds of CO_2_ and CH_4_ in vacuum,^51^ respectively). All other CH_*x*_ dissociation steps also present a lower energy barrier on Pt/HfC, and the dissociative adsorption of H_2_ is barrierless on Pt/HfC while it has an energy barrier of 0.49 eV on HfC. Despite H_2_ being a product and not a reactant in four of the five processes studied here, this result indicates that the TS for H_2_ formation on Pt/HfC will be 0.49 eV lower in energy (ignoring thermal contributions) compared to HfC, thus facilitating its production.

For methane reforming processes (i.e., DRM, SRM and POM), it is not possible to predict from the energy barriers alone what would be the dominant reaction pathway for CO production from CH_4_ (e.g., CH → C → CO or CH → CHO → CO), as this depends on an intricate interplay between all involved surface reaction and diffusion steps, and the interaction between the HfC and Pt sites. However, the analysis of the energy barriers suggests that Pt clusters should be responsible for CH_4_ and CO_2_ activation, while HfC should be responsible for H_2_O formation or dissociation. To bridge the gap between the atomic level and the macroscopic regime, DFT calculations can be coupled with KMC simulations which capture adsorbate mobility (diffusion) and coverage effects including those arising from lateral interactions.

### 3.2 KMC Results for the DRM Reaction

**Figure 3:**
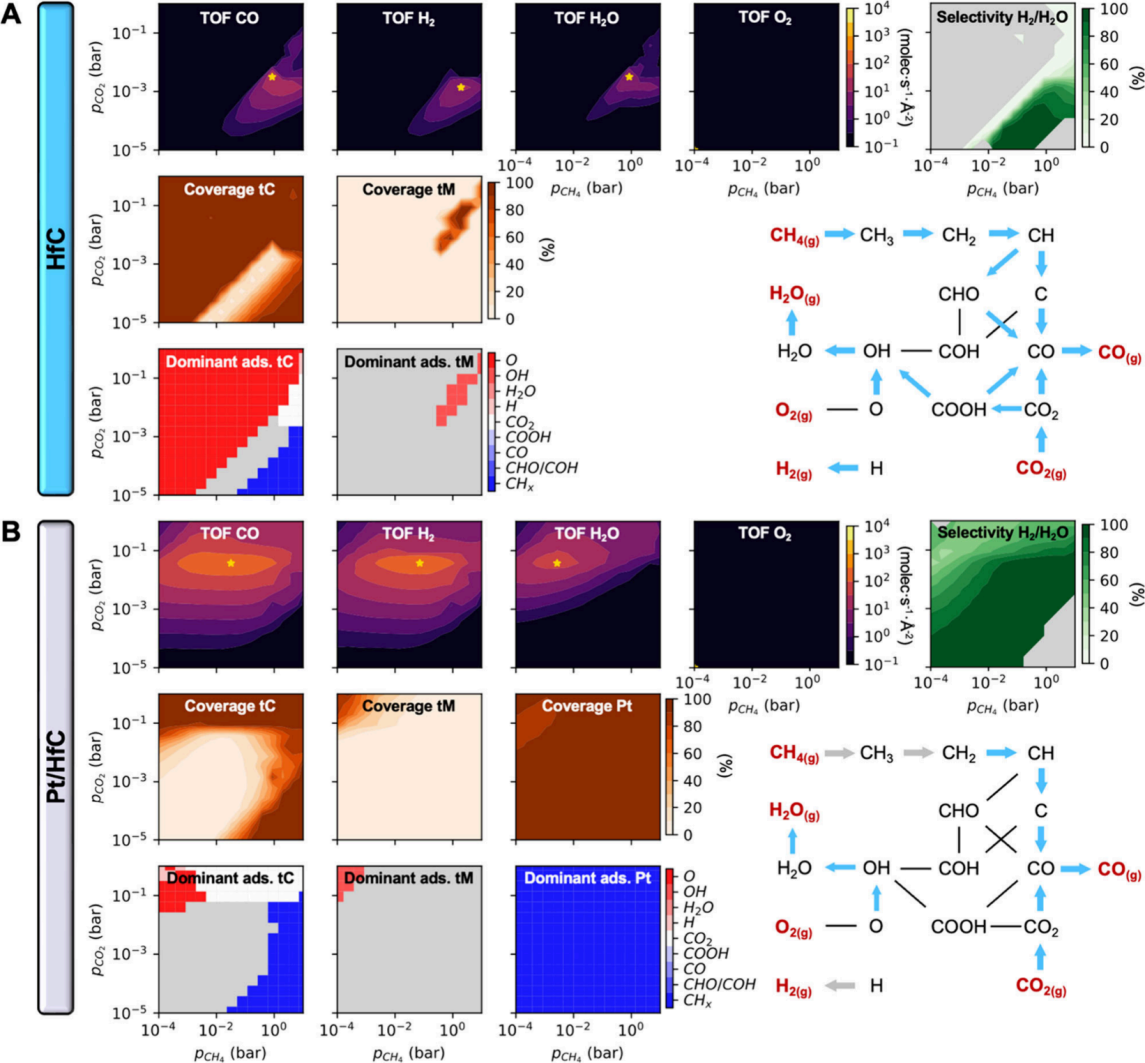
Computed TOF, H_2_/H_2_O selectivity, total surface coverage, kinetic phase diagrams and dominant reaction pathway for the DRM at 1000 K on (A) HfC and (B) Pt/HfC. In the TOF maps, black areas have a TOF < 10^–1^ molec·s^–1^·Å^–2^. In the selectivity maps, the selectivity is only computed if at least 200 product molecules have been formed; otherwise, it is shown in light gray. In the kinetic phase diagrams, nongray areas correspond to regions where the overall coverage is higher than 50%, and the color indicates the most abundant species at those conditions. The dominant reaction pathways correspond to the highest H_2_ TOF (golden ‘*’ marker in TOF maps) and are obtained from the process statistics plots in Figure S8 (HfC) and Figure S9 (Pt/HfC). Blue (HfC) and gray (Pt/HfC) arrows indicate what region is active for that step, and the arrow thickness is proportional to the event frequency. All heatmap plots are based on 225 (i.e., 15 × 15) KMC simulations at different (p_*CH*_4__*, p*_*CO*_2__) conditions.

We continue our discussion by analyzing the KMC results for the DRM activity on HfC first and then on Pt/HfC. The reaction temperature for the KMC simulations has been set to 1000 K, which is within the range of typical temperature conditions for the DRM process.^26^ For HfC, the contour lines in the CO TOF map overlap quite well with those of the H_2_O TOF map (Figure 3A). This means that the competing reaction CH_4_ + 3CO_2_ → 4CO + 2H_2_O, where water is produced instead of hydrogen, is more favored. As a result, the H_2_/H_2_O selectivity is quite low except at *p*_*CO*_2__ < 10^–4^ bar, where HfC is selective toward DRM but poorly active. The coverage heatmaps reveal that HfC is only active within a narrow *p*_*CO*_2__/*p*_*CH*_4__ ratio (i.e., in the range 0.01–0.001), provided that the total pressure is lower than ∼1 bar. Away from this optimal *p*_*CO*_2__/*p*_*CH*_4__ ratio, HfC is poisoned by O (too high) or CH_*x*_ (too low) species, resulting in no catalytic activity. The dominant reaction pathway at the conditions of the maximum H_2_ TOF involves mainly the formation of CO through C+O reaction or from direct CO_2_ dissociation, although there is also a small contribution from the CH → CHO → CO and the CO_2_ → COOH → CO alternative pathways (see reaction diagram in Figure 3A). There is however a significant production of H_2_O mainly coming from hydrogenation of O to H_2_O, which results in a moderate H_2_/H_2_O selectivity of 75%.

The deposition of Pt clusters on HfC, however, can boost the catalytic activity toward the DRM and shift the selectivity from H_2_O to H_2_, the desired product. Now, the CO and H_2_ TOF heatmaps overlap quite well except at *p*_*CO*_2__ > 0.1 bar (Figure 3B), where the formation of H_2_O is still more favored due to the higher concentration of O species. This shift from H_2_O to H_2_ production is reflected in the selectivity map and can be explained by the lower barrier for the H_2_ associative desorption on the Pt clusters, which facilitates the release of H_2_ as the main product before adsorbed H can react with O or OH to form H_2_O. At *p*_*CO*_2__ > 10^–1^ bar, however, a higher coverage of O species favors OH and H_2_O formation. Contrary to HfC, where the catalyst was only active within a narrow *p*_*CO*_2__/*p*_*CH*_4__ ratio, Pt/HfC is active in a wide range of operating conditions and *p*_*CH*_4__ can be reduced several orders of magnitude at a constant *p*_*CO*_2__ without a significant loss in catalytic activity. This is because Pt clusters can activate methane with practically no barrier (Figure 2). Still, Pt/HfC is not very active at *p*_*CH*_4__ > 1 bar due to poisoning of tC sites by CH_*x*_ species coming from the Pt clusters.

The maximum calculated TOF for DRM on Pt/HfC is about 230 H_2_·s^–1^·Å^–2^, almost 20 times higher than the maximum on HfC. The H_2_/H_2_O selectivity at these conditions is 95%, also higher than on HfC (75%). The horizontal contour lines in the TOF maps suggest that in most catalytically relevant conditions the activity is (at least in part) limited by the activation of CO_2_. The energy barrier for CO_2_ dissociation on the Pt clusters is only 0.64 eV (compared to 1.69 eV on HfC) but the clusters are always blocked by CH_*x*_ species (see kinetic phase diagrams in Figure 3B). This finding disagrees with the impression given from the DFT calculations, which suggest that the Pt clusters are responsible for CO_2_ dissociation. In fact, the reaction network diagram in Figure 3B reveals that, at least at the top of the H_2_ TOF heatmap, the Pt clusters do not contribute at all to this step. The analysis of the event frequencies for each elementary step (Figure S9) reveals that the role of the Pt clusters is to convert CH_4(g)_ to CH_2_ and form H_2(g)_, while the HfC region is responsible for the activation of CO_2(g)_ and conversion of CH_2_ to CO via C, allowing Pt/HfC to benefit from its bifunctional nature. This result also suggests that diffusion steps of CH_2_ from the Pt clusters to the HfC, and of H atoms from the HfC region to the Pt clusters are very important, allowing both regions of the catalyst to be connected.

### 3.3 Importance of Surface Diffusion and Lateral Interactions

Diffusion steps and lateral interactions between adsorbed species are sometimes overlooked but can play a crucial role in heterogeneous catalysis, so incorporating them in any kinetic modeling is necessary to obtain meaningful results. The reaction model used in this work includes diffusion steps for almost all adsorbed species within the Pt cluster, within the HfC region, and between the Pt clusters and the HfC region (Figure 1C). The only adsorbates whose diffusion has not been included are CO_2_ and COOH because, due to their larger size, their diffusion is predicted to be more hindered by the presence of other adsorbates. As for the lateral interactions, the cluster expansion used in this work includes 1NN two-body terms corresponding to all possible coadsorbed species (Tables S2 and S3) in the HfC region and in the Pt clusters. In this section, we analyze how the KMC results depend on these two important factors.

The impact of surface diffusion on the catalytic performance can be studied with KMC simulations by switching off such processes and rerunning the simulations. Let us begin by analyzing the importance of Pt↔HfC diffusion. The analysis of the event frequencies for the diffusion steps (Figure S9) reveals a substantial spillover of CH_2_ species from Pt to HfC. This alleviates the occupation of highly reactive Pt sites, making them available for new species to adsorb, such as CH_4_. Moreover, the HfC region, which is slow at activating CH_4(g)_, benefits from a continuous influx of CH_2_ species, thereby boosting the overall catalytic activity.

**Figure 4:**
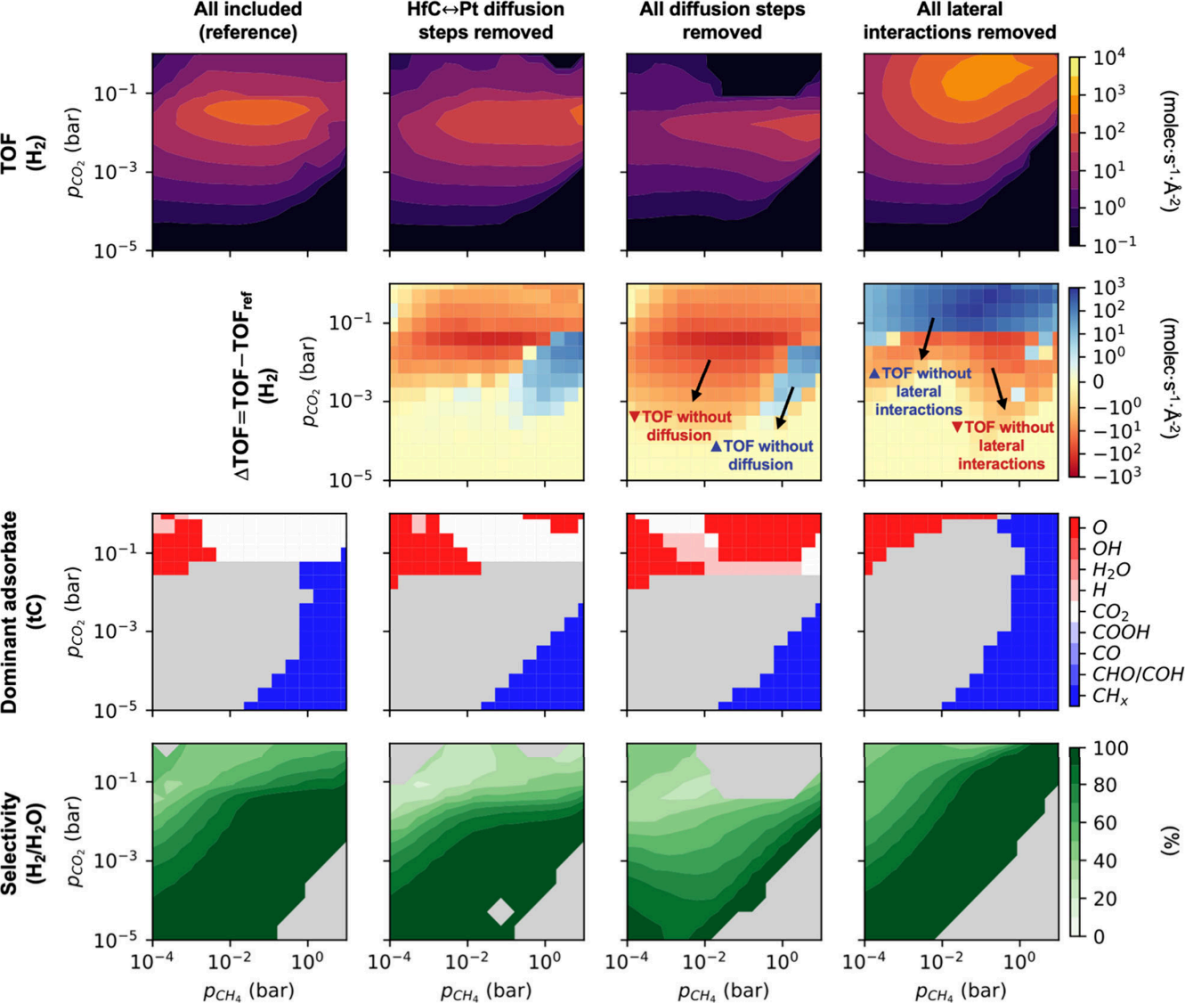
Computed TOF, ΔTOF, kinetic phase diagrams and selectivity heatmaps for the DRM on Pt/HfC at 1000 K under the following conditions (from left to right): diffusion steps and lateral interactions are included (i.e., reference), lateral interactions are included but HfC↔Pt diffusion steps are removed, lateral interactions are included but all diffusion steps are removed, and diffusion steps are included but lateral interactions are removed. In the TOF maps, black areas have a TOF < 10^–1^ molec·s^–1^·Å^–2^. In the ΔTOF maps, ΔTOF is computed as TOF-TOF_ref,_ where TOF_ref_ corresponds to the TOF for the simulation where diffusion steps and lateral interactions are included. In the kinetic phase diagrams, nongray areas correspond to regions where the overall coverage is higher than 50%, and the color indicates the most abundant species at those conditions. In the selectivity maps, the selectivity is only computed if at least 200 product molecules have been formed; otherwise, it is shown in light gray. All heatmap plots are based on 225 (i.e., 15 × 15) KMC simulations at different (*p*_*CH*_4__*, p*_*CO*_2__) conditions.

Figure 4 shows that removing HfC↔Pt diffusion steps cause the top of the H_2_ volcano plots to shift toward higher *p*_*CH*_4__ by several orders of magnitude, and the same shift is observed for the other products (Figure S5). The kinetic phase diagram shows that, without CH_2_ diffusion from Pt clusters to the HfC region, there is no poisoning of the tC sites by CH_*x*_ species, and Pt/HfC is now active at very high *p*_*CH*_4__. Moreover, the activity at moderate *p*_*CH*_4__ is lower compared to the reference simulation (i.e., with all diffusion steps), as the HfC region can no longer rely on Pt clusters to activate CH_4(g)_. These two factors explain the observed shift in the volcano plot.

Let us now consider the scenario where all diffusion steps are removed, that is, also disabling the diffusion within the Pt cluster and within the HfC region. Compared to the previous case, this change does not cause a further shift in the volcano plot; however, the overall activity is significantly reduced across all investigated (*p*_*CH*_4__*, p*_*CO*_2__) conditions. This general decrease is also clearly visible in the ΔTOF heatmaps and could be attributed to the presence of many bimolecular steps in the reaction model (e.g., C+O, CO_2_+H, O+H or OH+H), which depend on diffusion for the two reactants to meet. More importantly, removing the diffusion steps has a detrimental effect on the selectivity, shifting the production distribution from H_2_ to H_2_O. This selectivity shift can be explained by the inability of two H atoms to come into contact to form H_2_. Note that H species originate from CH_*x*_ dehydrogenation steps, but once a CH_4(g)_ molecule dissociates into C+4H, none of these 4 H species are 1NN. Instead, they are more likely to react with neighboring O or OH species, leading to the formation of H_2_O_(g)_.

Similarly, the influence of lateral interactions can be evaluated by switching them off and rerunning the KMC simulations. Figure 4 illustrates that, in the absence of lateral interactions, the peak of the volcano plot shifts significantly toward higher *p*_*CO*_2__. This shift occurs because, without lateral interactions, the tC sites are not poisoned by CO_2_ species. In the presence of lateral interactions, attractions between coadsorbed CO_2_–CO_2_ pairs (see Table S2) stabilize these adsorbed species and lead to the poisoning of tC sites, which dominate the lattice configuration at high *p*_*CO*_2__. Consequently, removing these interactions prevents CO_2_ poisoning, allowing the catalyst to remain active at more elevated *p*_*CO*_2__. Therefore, incorporating both diffusion and lateral interactions is essential for obtaining meaningful results.

### 3.4 Effect of Pt Loading and Temperature on DRM Activity

**Figure 5:**
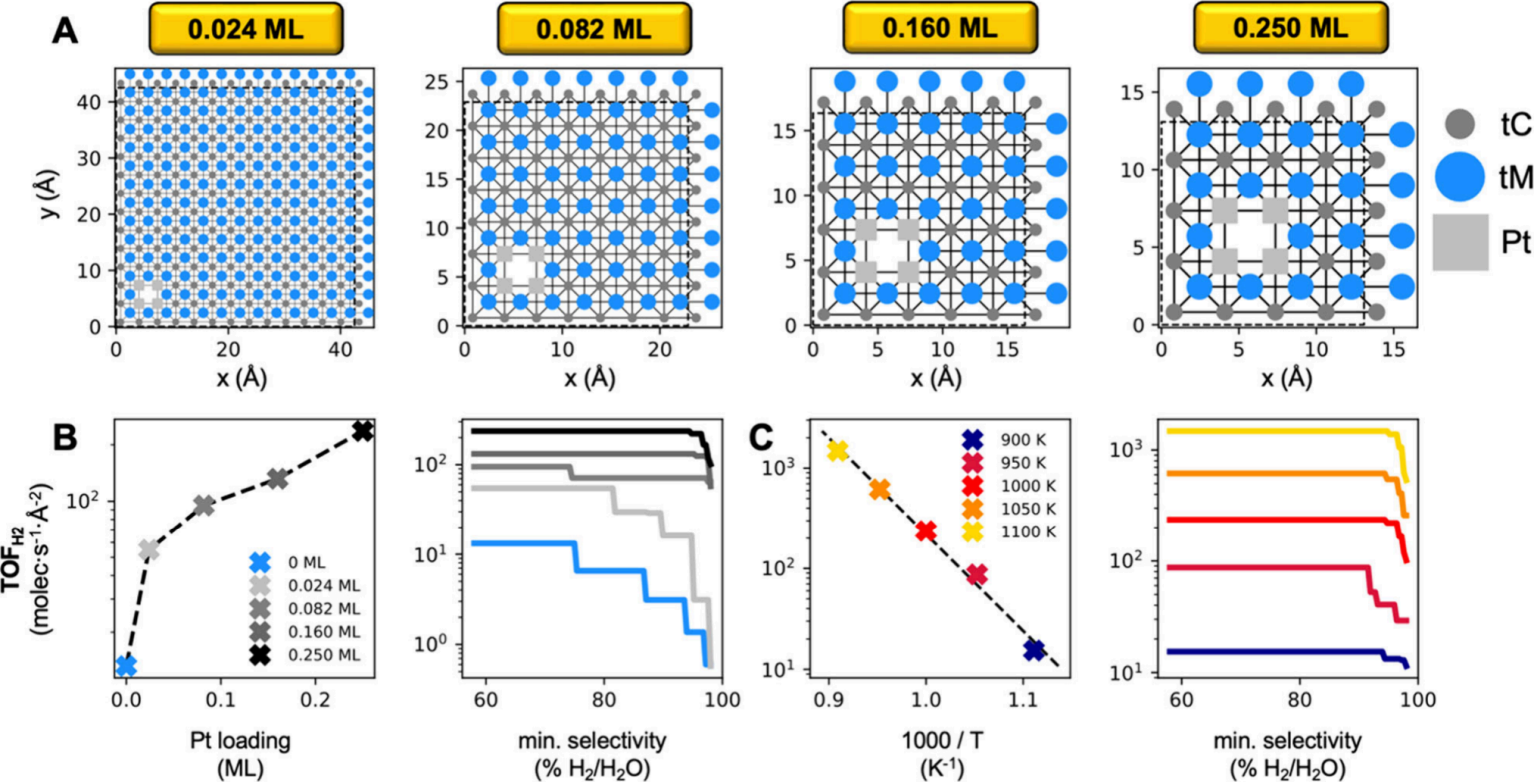
(A) KMC lattice models of Pt/HfC at various Pt loadings. (B) Maximum DRM TOF at 1000 K as a function of Pt loading (left panel) and as a function of a minimum H_2_/H_2_O selectivity for various Pt loadings (right panel). (C) Maximum TOF for DRM on Pt_0.25 ML_/HfC as a function of temperature (left panel) and minimum H_2_/H_2_O selectivity (right panel) for various temperatures. The dashed line represents a fit to the Arrhenius equation: ln*TOF* = ln *A* - *E*_*a*_/(*k*_*B*_*T*), where *A*, *E*_*a*_ and *k*_*B*_ are the pre-exponential, activation energy and Boltzmann constant, respectively. The linear regression yields *E*_*a*_ = 1.9 ± 0.1 eV, ln *A* = 27 ± 1, with *r*^2^ = 0.991.

All results for Pt/HfC discussed so far have been obtained by using a KMC lattice model corresponding to a Pt loading of 0.250 ML and at 1000 K. Now, let us evaluate the effect of Pt loading and reaction temperature on the DRM activity on Pt/HfC. To do so, we have run additional KMC simulations at 1000 K for three new lattice models corresponding to Pt loadings of 0.024, 0.082, and 0.160 ML (Figures 5A), as well as simulations at varying temperatures of 900, 950, 1050, and 1100 K for a Pt loading of 0.250 ML. Note that Pt loadings above 0.250 ML have not been considered, as they would likely lead to agglomeration or sintering, which would alter the catalyst structure and invalidate our DFT model. Furthermore, increasing the Pt loading reduces the noble metal utilization, which is undesirable from a cost-efficiency perspective.

We first focus on the effect of Pt loading. As shown in Figure 5B (left panel), the catalytic activity increases with Pt loading, exhibiting a sharp rise at very low loadings (<0.02 ML) and a more gradual increase at higher loadings. This trend can be rationalized by considering two distinct regimes. In the low-loading regime (0 to ∼0.02 ML), introducing Pt sites dramatically enhances the activity due to their nearly barrierless CH_4_ activation. At higher loadings, while adding more Pt still improves performance, its impact diminishes as CO_2_ activation on the HfC surface becomes more rate-limiting.

Regarding selectivity (Figure 5B, right panel, and Figure S6), increasing the Pt loading shifts the product distribution from H_2_O toward H_2_. The presence of Pt clusters promotes H_2_ formation from adsorbed hydrogen species, thus improving the overall H_2_/H_2_O ratio. Consequently, a Pt loading between approximately 0.08 and 0.16 ML provides the best balance between Pt utilization and catalytic performance. Notably, even a low Pt loading (∼0.02 ML) can boost the TOF by an order of magnitude at *p*_*CH*_4__ > 1 bar and by several orders of magnitude at lower *p*_*CH*_4__ pressures (Figure S6), underscoring the high specific metal utilization in this SCC.

Next, we analyze the effect of the reaction temperature. The results presented in Figure 5C reveal some important insights. An Arrhenius behavior is observed in the full range of temperatures studied, with an apparent activation energy of 1.9 eV. This indicates that a 100 K increase in reaction temperature results in approximately 1 order of magnitude increase in catalytic activity. The calculated activation energy value also reveals information about the potential rate-limiting step, as it is very close to the 2.08 eV free energy barrier for CO_2_ dissociation on HfC (see Gibbs free energy diagram for CO_2(g)_ activation on HfC in Figure S23). This free energy barrier has been calculated at 1000 K and *p*_*CO*_2__ = 3.72 × 10^–2^ bar, corresponding to *p*_*CO*_2__ at the peak of the H_2_ volcano plot, from where the data in Figure 5C is derived. This result suggests that CO_2_ dissociation can be the main or one of the rate-limiting steps. This conclusion is also supported by the analysis of the event frequencies in Figure S9, which shows that the formation of CO from CO_2_ on HfC only involves the direct dissociation of CO_2_ (and not the alternative COOH-mediated route), and with the contour lines in the TOF heatmaps, which are mainly perpendicular to the *p*_*CO*_2__ axis, implying that the adsorption and/or activation of CO_2_ is a controlling factor in the reaction kinetics.

The reaction temperature also moderately affects selectivity, as shown in the selectivity heatmaps (Figure S7). Increasing the temperature results in an increase of the selectivity toward H_2_. This improvement is likely due to the increased mobility of surface H species at higher temperatures, which facilitates their diffusion to Pt sites where they can desorb as H_2(g)_.

This concludes the analysis of the DRM on Pt/HfC. Based on the results discussed, further improvement in the catalytic performance toward the DRM could be achieved by incorporating additional active sites on the HfC region that are capable of dissociating CO_2_ with lower energy barriers. This could be achieved by the incorporation of surface C vacancies, which facilitate the dissociation of CO_2_.^52^ For instance, it has been shown by experiments and DFT calculations that C vacancies significantly enhance the catalytic performance of VC toward the RWGS reaction.

### 3.5 KMC Results for the SRM, POM, WGS, and RWGS Reactions

In this section, we assess the catalytic performance of HfC and Pt/HfC for other industrially relevant processes sharing the same reaction model. We begin by examining the other two methane reforming processes: SRM and POM. After that, we discuss the results for the WGS and the RWGS reactions.

**Figure 6:**
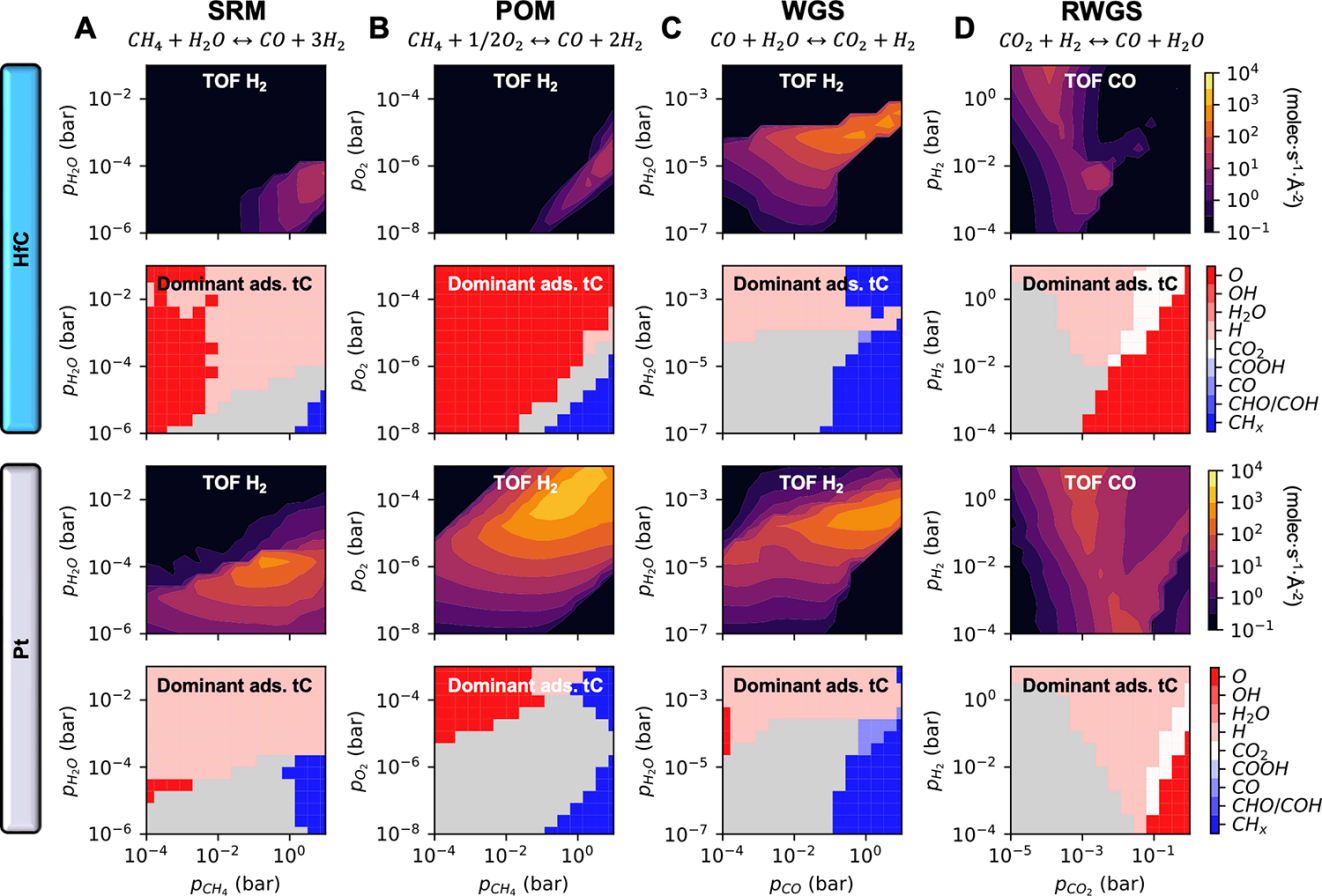
Computed TOF and phase diagrams for (A) SRM, (B) POM, (C) WGS and (D) RWGS on HfC and Pt/HfC at 1000 K. In the TOF maps, black areas have a TOF < 10^–1^ molec·s^–1^·Å^–2^. In the kinetic phase diagrams, nongray areas correspond to regions where the overall coverage is higher than 50%, and the color indicates the most abundant species at those conditions. The plots are based on 225 KMC simulations for each reaction at different (*p*_*A,*_*p*_*B*_) conditions.

The TOF heatmaps in Figure 6 reveal that bare HfC exhibits poor catalytic activity for both SRM and POM, even at the highest evaluated *p*_*CH*_4__ of 10 bar. Although higher TOFs might be achievable by further increasing *p*_*CH*_4__, this behavior is contrary to the case of DRM, where the highest H_2_ production occurs around *p*_*CH*_4__ ∼ 2 bar and at higher pressures due to poisoning of tC sites. The deposition of Pt clusters again boosts the catalytic activity and shifts the top of the volcano plots to *p*_*CH*_4__ values between 0.2 and 0.4 bar. Moreover, Pt clusters enable the reaction to proceed at significantly higher pressures of the oxidizing agent. For example, in POM at a fixed *p*_*CH*_4__ of 0.01 bar, the *p*_*O*_2__ at which tC sites become poisoned by O increases from 10^–8^ bar for HfC to 10^–5^ bar for Pt/HfC. This protection against O poisoning prevents the formation of an oxycarbide, as discussed later.

**Figure 7:**
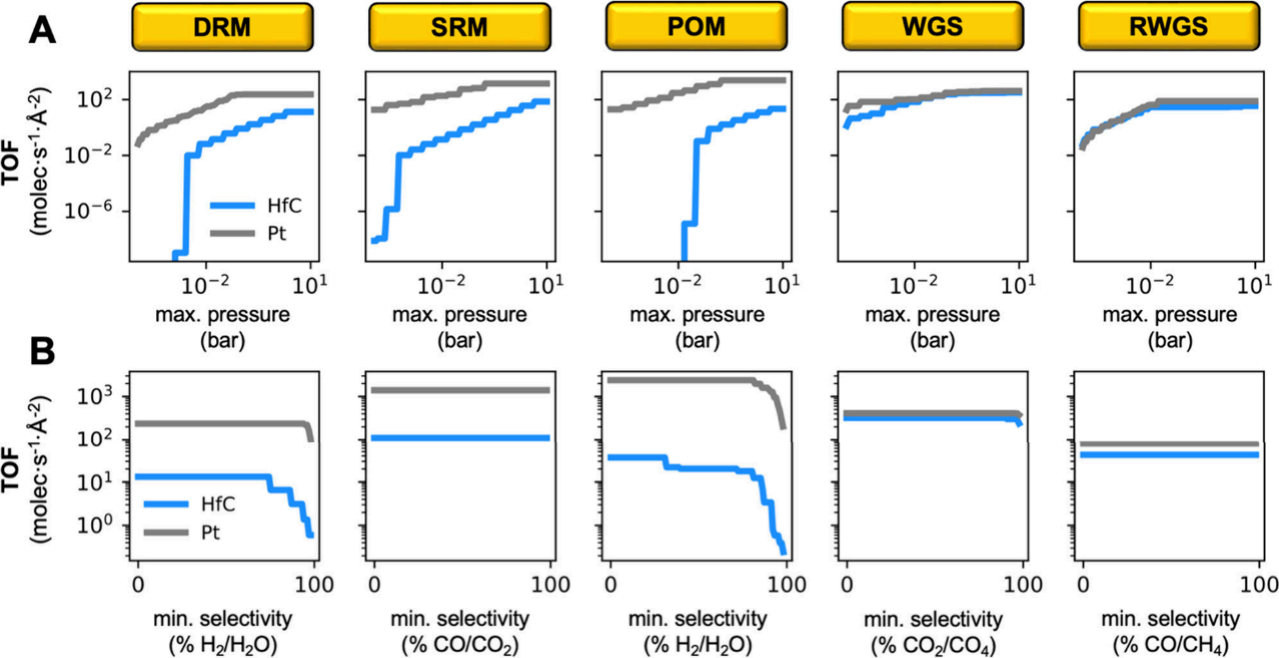
Maximum TOF values computed for DRM (H_2_), SRM (H_2_), POM (H_2_), WGS (H_2_) and RWGS (CO) reactions at 1000 K for (**A**) a given maximum pressure and (**B**) a given minimum selectivity, on HfC (blue) and Pt/HfC (gray).

The ability of Pt/HfC to catalyze the reaction at much lower pressures compared to HfC is clearly seen in Figure 7A. For both SRM and POM, the H_2_ production on Pt/HfC at a maximum total pressure of only ∼10^–4^ is comparable to that on bare HfC at a pressure of 10 bar. As for the selectivity, Figure 7B shows that for POM, the presence of Pt shifts the selectivity from H_2_O to H_2_, the same trend observed for DRM. Note that, for the SRM reaction, the H_2_/H_2_O selectivity is always 100% because water is the reactant, making selectivity toward H_2_ irrelevant. However, CO_2_ may compete with CO and as a side-product. This competition is generally not problematic, as syngas typically contains substantial amounts of CO_2_. In fact, the WGS reaction is often used in conjunction with the SRM to increase the H_2_/CO ratio in syngas by oxidizing CO to CO_2_.^53^

For the case of the WGS and RWGS reactions, however, the improvements in catalytic performance achieved by depositing Pt clusters on HfC are minimal, only moderately extending the range of operating pressures in which the catalyst is significantly active (i.e., TOF > 10^2^ molec·s^–1^·Å^–2^), as shown in Figure 6 and Figure 7. This limited effect is because the primary ability of Pt clusters is to dissociate CH_4(g)_ with negligible barrier, providing the HfC region with a continuous supply of CH_2_ species. While this is advantageous for methane reforming processes, it is not useful for the WGS and RWGS reactions. For the WGS, the analysis of the dominant reaction pathway (Figure S20) reveals that Pt clusters become poisoned by CO species, resulting in them having only a minor contribution to H_2_ formation. In the case of the RWGS, pathway analysis (Figure S21) shows that Pt clusters are poisoned by CH_*x*_ species. In this scenario, the only contribution of the Pt clusters is to the adsorption and dissociation of H_2_.

As a final remark, we note that both HfC and Pt/HfC are susceptible to O/OH poisoning under oxidative conditions, characterized by high partial pressures of O_2_, H_2_O, CO, and CO_2_. The high coverage of O and OH species can lead to the formation of an oxycarbide layer, where surface C atoms are replaced by O atoms, ultimately resulting in the oxidation of the carbide. Although this process cannot be modeled with our KMC framework, it is reasonable to assume that a HfC oxycarbide layer is formed at the conditions where the phase diagrams indicate O/OH poisoning.

### 3.6 Insights from KMC Simulations

At this stage, one might wonder how the TOF for DRM, SRM, POM, WGS and RWGS on HfC or Pt/HfC compares with experimental results from other catalysts reported in the literature. However, making such comparisons quantitatively in challenging for several reasons. First, many studies report different performance metrics, such as the conversion rate, the mass activity (measured in molecules produced per second and per mass of catalyst), or the geometric activity (measured in molecules produced per second and per geometric area of the catalyst). While mass or geometric activities provide useful insights, they do not directly reflect the intrinsic catalytic activity of a catalyst, which is defined as the number of molecules produced per second per unit of catalytically active area or site (TOF).^54^ TOF is the most scientifically relevant metric and is what can be estimated from KMC simulations.

Even when TOF is reported experimentally, making strict quantitative comparisons with simulation results is often not feasible. Real catalysts have defects, such as steps, edges, kinks, or vacancies, and the catalytic interface is dynamic,^55^ while the KMC simulations assume a pristine and static catalyst. In the case of supported particles, kinetic models are typically built assuming uniform cluster sizes and shapes, although real catalysts exhibit a distribution of particle sizes and shapes. Additional uncertainties are introduced by errors in the kinetic model parameters, such as the energy barriers (e.g., approximate exchange-correlation functionals in DFT calculations^56^), the rate constants (e.g., overestimation of rate constants in transition-state theory^57^), or the reaction model (e.g., omission of relevant elementary steps).

Despite the above-mentioned discrepancies between experimental conditions and simulation assumptions, KMC simulations remain an invaluable tool for capturing trends in activity and selectivity, which can guide the design and optimization of catalysts. In this study, KMC simulations demonstrated that both HfC and Pt/HfC should be active for all five reactions studied. For methane reforming processes, the deposition of Pt clusters is predicted to consistently improve the catalytic performance. Specifically, our simulations show that Pt clusters can increase the resistance of HfC to oxidation, significantly broadening the (*p*_*A,*_*p*_*B*_) range within which the catalyst remains active. Additionally, Pt clusters can shift the selectivity toward the desired product, as shown for DRM and POM. Moreover, Pt clusters consistently increase the TOF of the reaction across all (*p*_*A,*_*p*_*B*_) conditions in the simulations by providing different types of active sites that enable synergistic effects. They also make it possible to carry out the catalytic process at much lower partial pressures of reactants and reaction temperatures, which is important from a techno-economic perspective. For instance, for POM, the H_2_ production on Pt/HfC at *p*_*CH*_4__ of only 10^–4^ bar is equivalent to that on bare HfC at 10 bar.

Regarding the metal utilization, the KMC simulations reveal that the optimal catalytic performance is achieved at a Pt loading of 0.08–0.16 ML. However, even a Pt loading as low as ∼0.02 ML can boost the TOF by one or more orders of magnitude, depending on the operating conditions. Therefore, while a strict quantitative comparison of the predicted TOF with other catalysts is not possible, KMC simulations reveal that Pt/HfC has the potential to be an active, selective, and cost-effective catalyst for CO_2_ and CH_4_ conversion technologies, particularly for syngas production.

## 4 Conclusions

Our previous high-throughput DFT-based screening study identified Pt/HfC as the most promising TM/TMC combination for the conversion of CO_2_ and CH_4_. In this work, we verified the suitability of this SCC for the DRM, SRM, POM, WGS and RWGS reactions through KMC simulations. We have analyzed the interplay between the Pt clusters and the HfC support across a broad range of operating conditions (*p*_*A*_, *p*_*B*_, and *T*) and Pt loading, to identify the optimal conditions that maximize the activity and selectivity. The KMC simulations reveal that the deposition of Pt clusters consistently improves the catalytic performance of HfC for methane reforming processes by protecting it from oxidation, increasing the selectivity and TOF, and enabling the catalyst to remain active at much lower (*p*_*A*_, *p*_*B*_ and *T*) conditions with Pt loadings as low as ∼0.02 ML.

This work highlights the rich catalytic advantages of SCCs and underscores the importance of incorporating diffusion steps and lateral interactions in kinetic modeling, as these factors can play a dominant role. The systematic analysis presented in this work can be applied to explore the surface chemistry of other SCCs, which could revolutionize the field of heterogeneous catalysis, paving the way for the development of more efficient and sustainable industrial processes.

## Data Availability

A data set containing all relevant vasprun.xml files is available in the NOMAD repository^58^ with DOI 10.17172/NOMAD/2024.12.11-1. A collection of Python scripts that can generate the *Zacros* input files for all reactions studied here, analyze the data, and plot the results has been made available in the following public GitHub repository: https://github.com/hprats/KMC_MethaneReforming_PtHfC.

## Supporting Information Available

Additional details on the KMC models; figures showing the results for the several tests (i.e., lattice size, number of grid points, random seeds, diffusion steps, lateral interactions, Pt loading and reaction temperature), process statistics plots, calculation of rate constants, use of the BEP relations, and ensuring that the steady-state is reached (PDF)
