# Supplementary material for: First-Principles Kinetic Monte Carlo Simulations for Single-Cluster Catalysis: Study of CO2 and CH4 Conversion on Pt/HfC
Source: ACS Catal. 2025 Feb 3;15(4):2904–15. doi: 10.1021/acscatal.4c07877 (PMC11851442; doi:10.1021/acscatal.4c07877)
Supplement: Supplementary file 1 — cs4c07877_si_001.pdf [file cs4c07877_si_001.pdf]

First-principles Kinetic Monte Carlo simulations for single-cluster catalysis: Study of CO_2 and CH_4 conversion on Pt/HfC

Hector Prats*[a,b] and Michail Stamatakis[c]

[a] *Department of Chemistry, Physical & Theoretical Chemistry Laboratory, University of Oxford, South Parks Road, Oxford OX1 3QZ, UK*

[b] *Institute of Materials Chemistry, Technische Universität Wien, 1060 Vienna, Austria*

[c] *Department of Chemistry, Inorganic Chemistry Lab, University of Oxford, Oxford OX1 3QR, UK*

*Corresponding author: Hector Prats (hector.prats@tuwien.ac.at)

Supporting Tables 2

Supporting Figures 5

Supporting Notes 29

References 32

Supporting Tables

Table S1. List of steps included in the KMC reaction model with the calculated forward energy barrier ($\Delta E^{\neq,fwd}$), reverse energy barrier ($\Delta E^{\neq,rev}$) and reaction energy (ΔE^{reac}). The zero-point energy term is not included. When two or more reactant or product species are involved, $\Delta E^{\neq}$ and ΔE^{reac} are calculated at infinite separation (i.e., not including the lateral interaction). In the process with ID aH$_2$_Pt, $\&^{Pt}$ means an empty Pt site or occupied by any adsorbed species. All numbers are rounded to 2 decimals. To speed-up the KMC simulations, all symmetric diffusions with a barrier lower than 0.40 eV have been set to 0.40 eV.

ID	Step	$\Delta E^{\neq,fwd}\ (eV)$	$\Delta E^{\neq,rev}\ (eV)$	$\Delta E^{reac}\ (eV)$
aCH$_4$_HfC	$CH_{4(g)} + 2 *^{tC} \rightleftharpoons CH_3^{*tC} + H^{*tC}$	0.948	1.232	-0.284
aCH$_4$_Pt	$CH_{4(g)} + 3 *^{Pt} \rightleftharpoons CH_3^{*2Pt} + H^{*Pt}$	0.029	1.145	-1.116
aCH$_4$_in	$CH_{4(g)} + 2 *^{Pt} + *^{tC} \rightleftharpoons CH_3^{*2Pt} + H^{*tC}$	0.029	0.854	-0.825
aO$_2$_HfC	$O_{2(g)} + 2 *^{tC} \rightleftharpoons 2O^{*tC}$	0.0	5.265	-5.265
aO$_2$_Pt	$O_{2(g)} + 2 *^{Pt} \rightleftharpoons 2O^{*Pt}$	0.0	4.426	-4.426
aCO_HfC	$CO_{(g)} + *^{tC} \rightleftharpoons CO^{*tC}$	0.0	1.724	-1.724
aCO_Pt	$CO_{(g)} + *^{Pt} \rightleftharpoons CO^{*Pt}$	0.0	2.477	-2.477
aH$_2$_HfC	$H_{2(g)} + 2 *^{tC} \rightleftharpoons 2H^{*tC}$	0.494	1.645	-1.151
aH$_2$_Pt	$H_{2(g)} + 2 *^{Pt} \rightleftharpoons 2H^{*Pt}$	0.0	1.733	-1.733
aH$_2$_in	$H_{2(g)} + *^{Pt} + *^{tC} \rightleftharpoons H^{*Pt} + H^{*tC}$	0.0	1.442	-1.442
aH$_2$_in_CH	$H_{2(g)} + CH^{*4Pt} + 2 *^{tC} \rightleftharpoons CH^{*4Pt} + 2H^{*tC}$	0.0	1.151	-1.151
aH$_2$_in_C	$H_{2(g)} + C^{*4Pt} + 2 *^{tC} \rightleftharpoons C^{*4Pt} + 2H^{*tC}$	0.0	1.151	-1.151
aCO$_2$_HfC	$CO_{2(g)} + *^{tC} \rightleftharpoons CO_2^{*tC}$	0.0	1.572	-1.572
aCO$_2$_Pt	$CO_{2(g)} + 3 *^{Pt} \rightleftharpoons CO_2^{*3Pt}$	0.0	1.355	-1.355
aH$_2$O_HfC	$H_2O_{(g)} + *^{tM} \rightleftharpoons H_2O^{*tM}$	0.0	0.789	-0.789
aH$_2$O_Pt	$H_2O_{(g)} + *^{Pt} \rightleftharpoons H_2O^{*Pt}$	0.0	0.789	-0.789
bCH$_3$_HfC	$CH_3^{*tC} + *^{tC} \rightleftharpoons CH_2^{*tC} + H^{*tC}$	0.880	0.834	0.045
bCH$_3$_Pt	$CH_3^{*2Pt} + *^{Pt} \rightleftharpoons CH_2^{*2Pt} + H^{*Pt}$	0.467	0.374	0.092
bCH$_3$_in	$CH_3^{*2Pt} + *^{tC} \rightleftharpoons CH_2^{*2Pt} + H^{*tC}$	0.467	0.083	0.384
bCH$_2$_HfC	$CH_2^{*tC} + *^{tC} \rightleftharpoons CH^{*tC} + H^{*tC}$	0.725	0.658	0.066
bCH$_2$_in	$CH_2^{*2Pt} + 2 *^{Pt} + *^{tC} \rightleftharpoons CH^{*4Pt} + H^{*tC}$	0.511	0.435	0.076
bCH_HfC	$CH^{*tC} + *^{tC} \rightleftharpoons C^{*tC} + H^{*tC}$	1.284	0.971	0.313

bCH_in	$CH^{*4Pt} + *^{tC} \rightleftharpoons C^{*4Pt} + H^{*tC}$	0.853	0.801	0.053
fCO_HfC	$C^{*tC} + O^{*tC} \rightleftharpoons CO^{*tC} + *^{tC}$	1.290	2.176	-0.886
fCO_in	$C^{*4Pt} + O^{*tC} \rightleftharpoons CO^{*Pt} + 3*^{Pt} + *^{tC}$	1.670	2.857	-1.187
CHtoCHO_HfC	$CH^{*tC} + O^{*tC} \rightleftharpoons CHO^{*tC} + *^{tC}$	1.375	1.781	-0.406
CHtoCHO_in	$CH^{*4Pt} + O^{*tC} \rightleftharpoons CHO^{*2Pt} + 2*^{Pt} + *^{tC}$	1.463	1.883	-0.420
CHOtoCO_HfC	$CHO^{*tC} + *^{tC} \rightleftharpoons CO^{*tC} + H^{*tC}$	1.295	1.463	-0.167
CHOtoCO_Pt	$CHO^{*2Pt} + *^{Pt} \rightleftharpoons CO^{*Pt} + *^{Pt} + H^{*Pt}$	0.938	1.943	-1.005
CHOtoCO_in	$CHO^{*2Pt} + *^{tC} \rightleftharpoons CO^{*Pt} + *^{Pt} + H^{*tC}$	0.938	1.652	-0.714
CtoCOH_HfC	$C^{*tC} + OH^{tM} \rightleftharpoons COH^{*tC} + *^{tM}$	0.460	0.796	-0.336
CtoCOH_in	$C^{*4Pt} + OH^{*tM} \rightleftharpoons COH^{*2Pt} + 2*^{Pt} + *^{tM}$	0.373	0.334	0.039
COHtoCHO_HfC	$COH^{*tC} \rightleftharpoons CHO^{*tC}$	1.556	2.352	-0.796
COHtoCHO_Pt	$COH^{*2Pt} \rightleftharpoons CHO^{*2Pt}$	0.865	1.790	-0.925
bCO_2_HfC	$CO_2^{*tC} + *^{tC} \rightleftharpoons CO^{*tC} + O^{*tC}$	1.693	1.216	0.477
bCO_2_Pt	$CO_2^{*3Pt} + *^{Pt} \rightleftharpoons *^{Pt} + CO^{*Pt} + O^{*Pt}$	0.642	0.716	-0.074
bCO_2_in	$CO_2^{*3Pt} + *^{tC} \rightleftharpoons CO^{*Pt} + 2*^{Pt} + O^{*tC}$	1.038	1.532	-0.494
fOH_HfC	$O^{*tC} + H^{*tC} + *^{tM} \rightleftharpoons 2*^{tC} + OH^{*tM}$	1.320	0.906	0.414
fOH_Pt	$O^{*2Pt} + H^{*Pt} \rightleftharpoons OH^{*2Pt} + *^{Pt}$	0.921	1.269	-0.348
fOH_in_a	$O^{*2Pt} + H^{*tC} \rightleftharpoons OH^{*2Pt} + *^{tC}$	1.029	1.668	-0.639
fOH_in_b	$O^{*2Pt} + *^{tM} + H^{*tC} \rightleftharpoons 2*^{Pt} + OH^{*tM} + *^{tC}$	0.557	0.563	-0.006
fH_2O_HfC	$OH^{*tM} + H^{*tC} \rightleftharpoons H_2O^{*tM} + *^{tC}$	0.306	0.255	0.050
fH_2O_Pt	$OH^{*2Pt} + H^{*Pt} \rightleftharpoons H_2O^{*Pt} + 2*^{Pt}$	1.513	0.539	0.974
fH_2O_in	$OH^{*2Pt} + H^{*tC} \rightleftharpoons *^{Pt} + H_2O^{*Pt} + *^{tC}$	1.666	0.983	0.683
CO2toCOOH_HfC	$CO_2^{*tC} + H^{*tC} \rightleftharpoons COOH^{*tC} + *^{tC}$	1.301	0.667	0.634
CO2toCOOH_Pt	$CO_2^{*3Pt} + H^{*Pt} \rightleftharpoons COOH^{*2Pt} + 2*^{Pt}$	1.924	1.552	0.372
CO2toCOOH_in	$CO_2^{*3Pt} + H^{*tC} \rightleftharpoons COOH^{*2Pt} + *^{Pt} + *^{tC}$	1.633	1.552	0.081
COOHtoCO_HfC	$COOH^{*tC} + *^{tM} \rightleftharpoons CO^{*tC} + OH^{*tM}$	0.469	0.213	0.256
COOHtoCO_Pt	$COOH^{*2Pt} + *^{Pt} \rightleftharpoons CO^{*Pt} + OH^{*2Pt}$	1.038	1.833	-0.794
COOHtoCO_in	$COOH^{*2Pt} + *^{tM} \rightleftharpoons *^{Pt} + CO^{*Pt} + OH^{*tM}$	1.038	1.200	-0.161
dO_HfC	$O^{*tC} + *^{tC} \rightleftharpoons *^{tC} + O^{*tC}$	1.221	1.221	0.0
dO_Pt	$O^{*2Pt} + *^{Pt} \rightleftharpoons *^{Pt} + O^{*2Pt}$	0.176	0.176	0.0
dO_in	$O^{*2Pt} + *^{tC} \rightleftharpoons 2*^{Pt} + O^{*tC}$	0.509	0.928	-0.420

dH_HfC	$H^{*tC} + *^{tC} \rightleftharpoons *^{tC} + H^{*tC}$	1.179	1.179	0.0
dH_Pt	$H^{*Pt} + *^{Pt} \rightleftharpoons *^{Pt} + H^{*Pt}$	0.126	0.126	0.0
dH_in	$H^{*Pt} + *^{tC} \rightleftharpoons *^{Pt} + H^{*tC}$	0.993	0.702	0.291
dCO_HfC	$CO^{*tC} + *^{tC} \rightleftharpoons *^{tC} + CO^{*tC}$	1.156	1.156	0.0
dCO_Pt	$CO^{*Pt} + *^{Pt} \rightleftharpoons *^{Pt} + CO^{*Pt}$	0.547	0.547	0.0
dCO_in	$CO^{*Pt} + *^{tC} \rightleftharpoons *^{Pt} + CO^{*tC}$	1.730	0.977	0.754
dOH_HfC	$OH^{*tM} + *^{tM} \rightleftharpoons *^{tM} + OH^{*tM}$	0.279	0.279	0.0
dOH_Pt	$OH^{*2Pt} + *^{Pt} \rightleftharpoons *^{Pt} + OH^{*2Pt}$	0.371	0.371	0.0
dOH_in	$OH^{*2Pt} + *^{tM} \rightleftharpoons 2 *^{Pt} + OH^{*tM}$	0.966	0.333	0.633
dH$_2$O_HfC	$H_2O^{*tM} + *^{tM} \rightleftharpoons *^{tM} + H_2O^{*tM}$	0.387	0.387	0.0
dH$_2$O_Pt	$H_2O^{*Pt} + *^{Pt} \rightleftharpoons *^{Pt} + H_2O^{*Pt}$	0.221	0.221	0.0
dH$_2$O_in	$H_2O^{*Pt} + *^{tM} \rightleftharpoons *^{Pt} + H_2O^{*tM}$	0.520	0.520	0.0
dCH$_3$_HfC	$CH_3^{*tC} + *^{tC} \rightleftharpoons *^{tC} + CH_3^{*tC}$	1.268	1.268	0.0
dCH$_3$_Pt	$CH_3^{*2Pt} + *^{Pt} \rightleftharpoons *^{Pt} + CH_3^{*2Pt}$	0.138	0.138	0.0
dCH$_3$_in	$CH_3^{*2Pt} + *^{tC} \rightleftharpoons 2 *^{Pt} + CH_3^{*tC}$	2.192	1.651	0.541
dCH$_2$_HfC	$CH_2^{*tC} + *^{tC} \rightleftharpoons *^{tC} + CH_2^{*tC}$	1.277	1.277	0.0
dCH$_2$_Pt	$CH_2^{*2Pt} + *^{Pt} \rightleftharpoons *^{Pt} + CH_2^{*2Pt}$	0.244	0.244	0.0
dCH$_2$_in	$CH_2^{*2Pt} + *^{tC} \rightleftharpoons 2 *^{Pt} + CH_2^{*tC}$	0.937	0.734	0.203
dCH_HfC	$CH^{*tC} + *^{tC} \rightleftharpoons *^{tC} + CH^{*tC}$	0.687	0.687	0.0
dCH_in	$CH^{*4Pt} + *^{tC} \rightleftharpoons 4 *^{Pt} + CH^{*tC}$	1.426	1.233	0.193
dC_HfC	$C^{*tC} + *^{tC} \rightleftharpoons *^{tC} + C^{*tC}$	1.449	1.449	0.0
dCHO_HfC	$CHO^{*tC} + *^{tC} \rightleftharpoons *^{tC} + CHO^{*tC}$	1.532	1.532	0.0
dCHO_Pt	$CHO^{*2Pt} + *^{Pt} \rightleftharpoons *^{Pt} + CHO^{*2Pt}$	0.416	0.416	0.0
dCHO_in	$CHO^{*2Pt} + *^{tC} \rightleftharpoons 2 *^{Pt} + CHO^{*tC}$	1.461	1.254	0.207
dCOH_HfC	$COH^{*tC} + *^{tC} \rightleftharpoons *^{tC} + COH^{*tC}$	1.288	1.288	0.0
dCOH_Pt	$COH^{*2Pt} + *^{Pt} \rightleftharpoons *^{Pt} + COH^{*2Pt}$	0.192	0.192	0.0
dCOH_in	$COH^{*2Pt} + *^{tC} \rightleftharpoons 2 *^{Pt} + COH^{*tC}$	1.372	1.295	0.077

Table S2. Formation energies (in eV) of the gas-phase and surface species included in the energetics model for the KMC simulations. The formation energies for the adsorbed species correspond to the single-body terms included in the cluster expansion.

	CH_4	O_2	CH_3	CH_2	CH	C	H	O	OH	H_2O	CO	CO_2	CHO	COH	COOH
Gas	0.000	2.613								-1.224	1.954	0.000			
tC			0.292	0.913	1.555	2.443	-0.576	-1.326			0.231	-1.527	-0.177	0.619	-1.513
tM									-1.488	-2.103					
Pt			-0.250	0.710	1.362	1.990	-0.867	-0.906	-2.121	-2.013	-0.523	-1.355	-0.384	0.541	-1.850

Table S3. Lateral interactions (in eV) included in the KMC model for coadsorbed species on the HfC region (i.e., adsorbed on tC or tM sites). They correspond to the two-body terms included in the cluster expansion.

	CH_3	CH_2	CH	C	H	O	OH	H_2O	CO	CO_2	CHO	COH	COOH
CH_3	0.212	0.041	-0.014	0.022	-0.014	0.034	-0.265	-0.072	0.057	-0.362	0.014	0.010	0.016
CH_2		-0.120	-0.104	-0.139	0.006	-0.079	-0.220	0.003	-0.055	-0.217	-0.050	0.017	-0.023
CH			-0.058	-0.022	0.004	-0.041	-0.118	-0.023	0.002	-0.240	0.010	-0.006	0.029
C				0.093	0.044	-0.036	-0.186	-0.019	0.117	-0.167	0.043	0.033	0.033
H					0.038	-0.083	-0.272	-0.077	0.069	-0.266	0.024	0.009	-0.078
O						0.033	-0.204	-0.041	-0.033	-0.161	-0.031	0.094	-0.024
OH							-0.087	-0.042	-0.239	-0.106	-0.141	-0.083	-0.124
H_2O								-0.035	-0.023	-0.030	-0.033	0.001	-0.023
CO									0.177	-0.183	0.079	0.011	0.053
CO_2										-0.144	0.052	0.084	-0.135
CHO											-0.063	0.017	0.037
COH												0.057	0.098
COOH													-0.070

Table S4. Lateral interactions (in eV) included in the KMC model for coadsorbed species on the Pt clusters. They correspond to the two-body terms included in the cluster expansion. Those cases where the coadsorption is not possible have been labelled as N/A and highlighted in yellow. For instance, the coadsorption of CH_3 and CO_2 is not possible because CH_3 occupies 2 Pt sites and CO_2 occupies 3 Pt sites, but each Pt cluster only contains 4 Pt sites. For CH and C, no coadsorption is possible, as this species occupy all 4 Pt sites in the cluster.

	CH_3	CH_2	CH	C	H	O	OH	H_2O	CO	CO_2	CHO	COH	COOH
CH_3	0.032	0.111	N/A	N/A	0.072	0.002	0.001	-0.083	-0.020	N/A	0.135	0.257	0.082
CH_2		0.259	N/A	N/A	0.154	0.195	0.092	-0.262	0.053	N/A	0.215	0.208	0.233
CH			N/A	N/A	N/A	N/A	N/A	N/A	N/A	N/A	N/A	N/A	N/A
C				N/A	N/A	N/A	N/A	N/A	N/A	N/A	N/A	N/A	N/A
H					0.376	0.122	0.075	-0.174	0.029	0.751	0.197	0.285	0.157
O						0.183	0.083	-0.050	0.018	N/A	0.224	0.194	0.171
OH							-0.165	0.003	-0.036	N/A	0.090	0.216	0.044
H_2O								-0.151	-0.107	-0.656	-0.357	-0.208	-0.351
CO									0.029	0.163	-0.020	0.058	-0.038
CO_2										N/A	N/A	N/A	N/A
CHO											0.278	0.301	0.240
COH												0.378	0.330
COOH													0.224

Supporting Figures

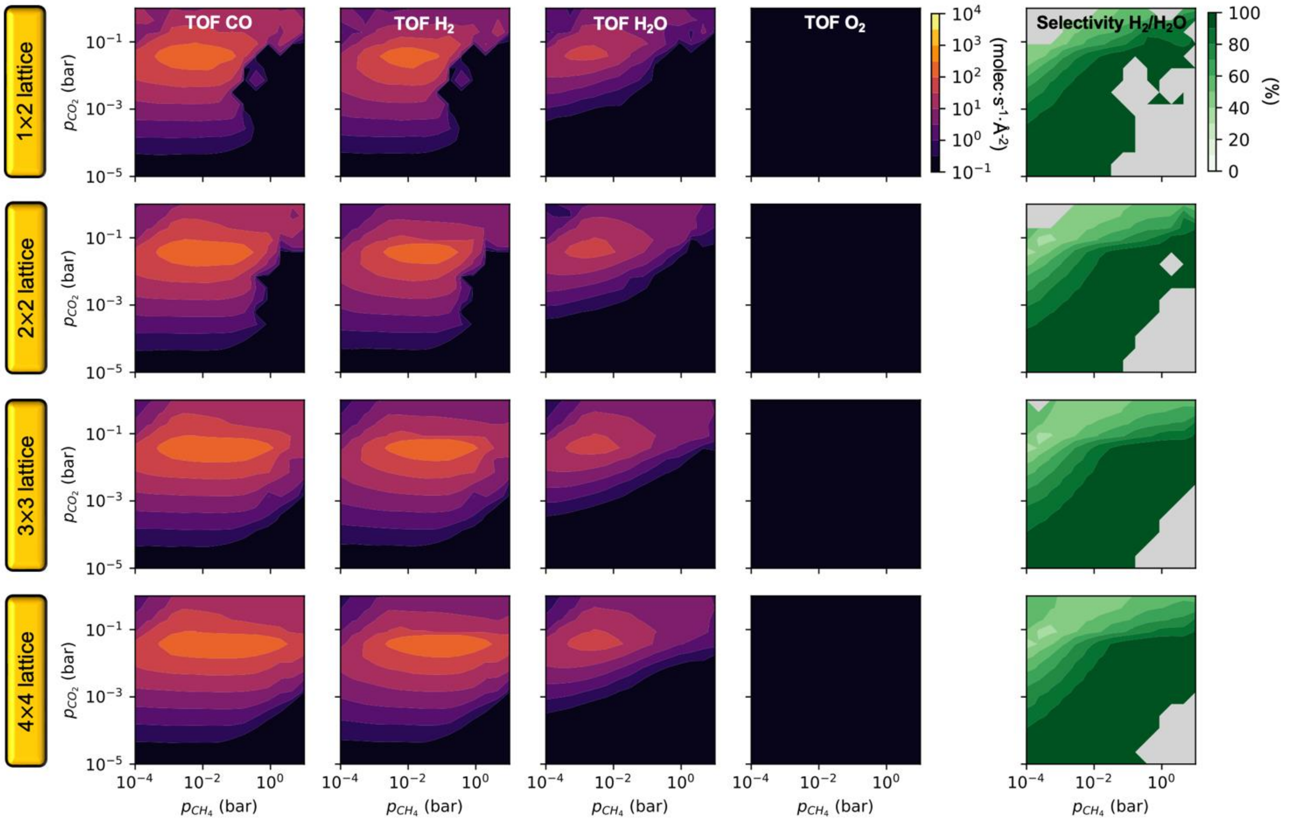

Figure S1. Computed TOF and H_2/H_2O selectivity for the DRM at 1000 K on Pt/HfC as a function of the lattice size (i.e., $N \times N$, where N is the number of repetitions / tilings along the a or b directions of the unit cell). In the TOF maps, black areas have a TOF < 10^{-1} molec·s^{-1}·Å^{-2}. In the selectivity maps, the selectivity is only computed if at least 200 product molecules have been formed; otherwise, it is shown in light grey. All heatmap plots are based on 225 (i.e., 15×15) KMC simulations at different (p_{CH_4}, p_{CO_2}) conditions.

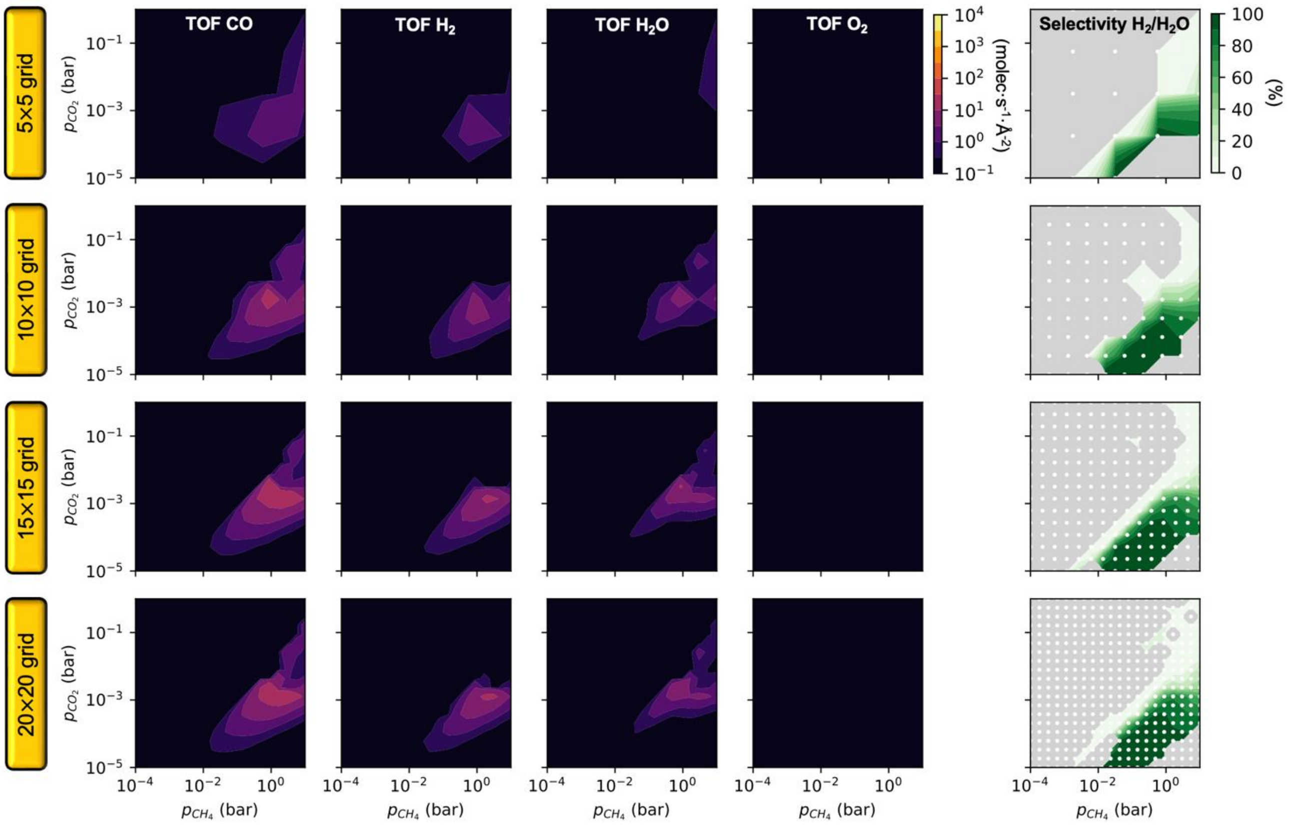

Figure S2. Computed TOF and H_2/H_2O selectivity for the DRM at 1000 K on HfC using 4 different logarithmically spaced grid of $N \times N$ points to sample the (p_{CH_4}, p_{CO_2}) space, where $N = 5$, 10, 15 or 20. In the TOF maps, black areas have a TOF < 10^{-1} molec·s^{-1}·Å^{-2}. In the selectivity maps, the selectivity is only computed if at least 200 product molecules have been formed; otherwise, it is shown in light grey. The grid points used for the sampling are shown as white dots in the selectivity maps.

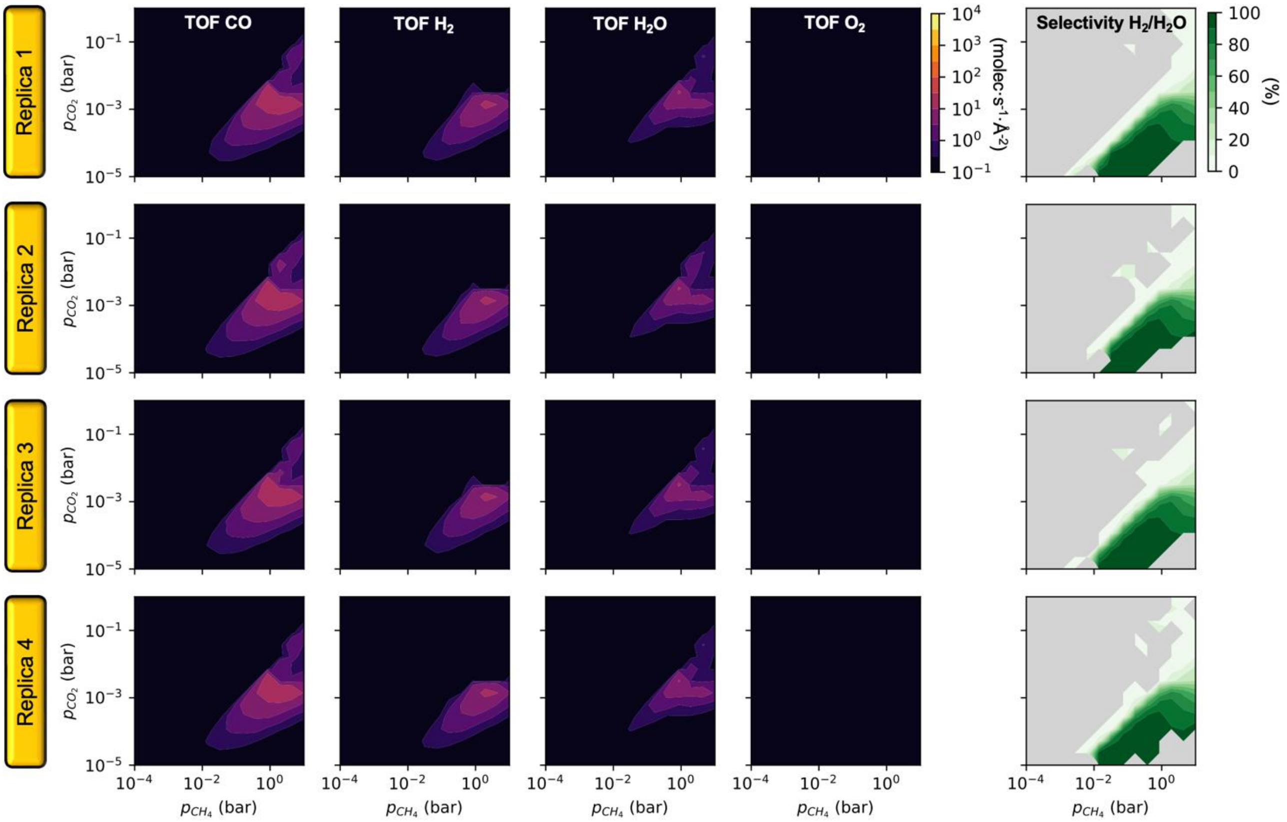

Figure S3. Computed TOF and H_2/H_2O selectivity for the DRM at 1000 K on HfC obtained from 4 KMC replicas with different initial random seed. In the TOF maps, black areas have a TOF < 10^{-1} molec·s^{-1}·$Å^{-2}$. In the selectivity maps, the selectivity is only computed if at least 200 product molecules have been formed; otherwise, it is shown in light grey. All heatmap plots are based on 225 (i.e., 15×15) KMC simulations at different (p_{CH_4}, p_{CO_2}) conditions.

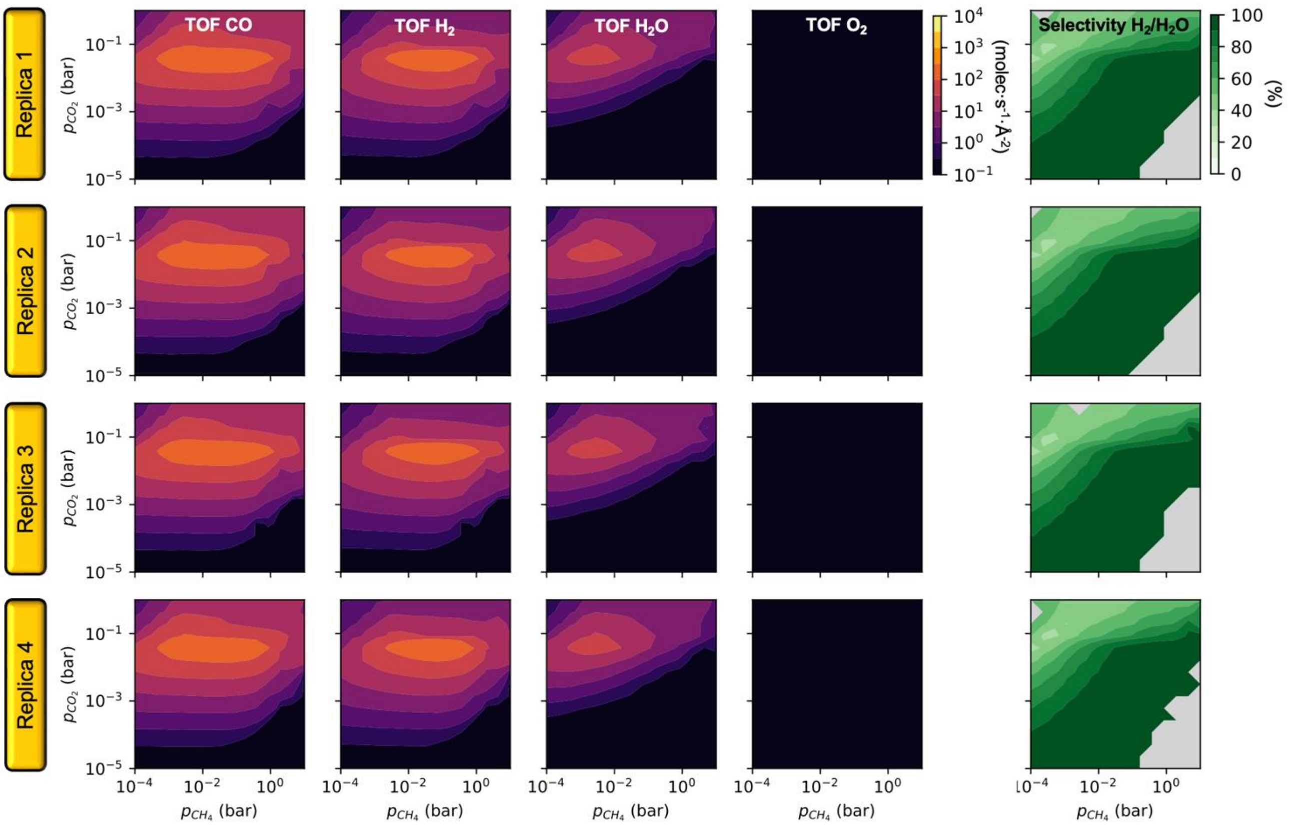

Figure S4. Computed TOF and H_2/H_2O selectivity for the DRM at 1000 K on Pt/HfC obtained from 4 KMC replicas with different initial random seed. In the TOF maps, black areas have a TOF < 10^{-1} molec·s^{-1}·Å^{-2}. In the selectivity maps, the selectivity is only computed if at least 200 product molecules have been formed; otherwise, it is shown in light grey. All heatmap plots are based on 225 (i.e., 15×15) KMC simulations at different (p_{CH_4}, p_{CO_2}) conditions.

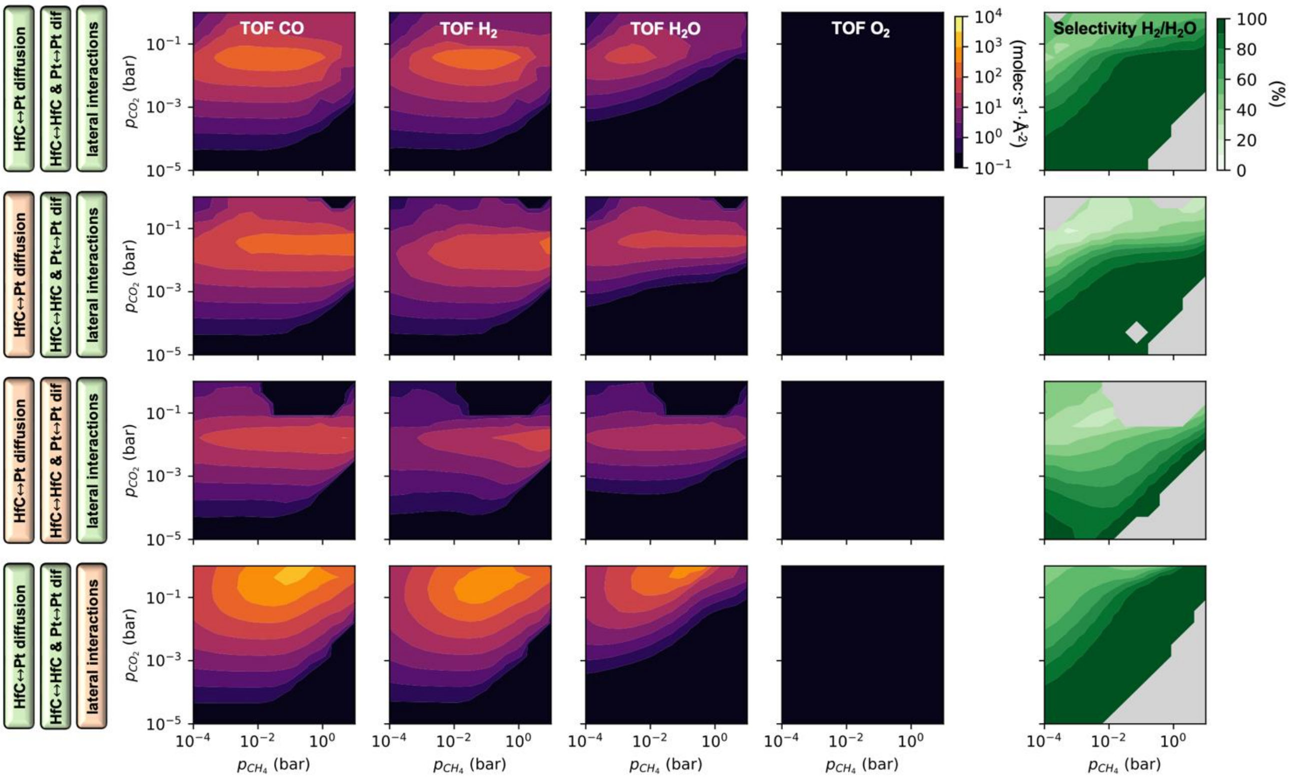

Figure S5. Computed TOF and H_2/H_2O selectivity for the DRM at 1000 K on Pt/HfC under the following conditions (from top to bottom): diffusion steps and lateral interactions are included, lateral interactions are included but HfC↔Pt diffusion steps are removed, lateral interactions are included but all diffusion steps are removed, and diffusion steps are included but lateral interactions are removed. In the TOF maps, black areas have a TOF < 10^{-1} molec·s^{-1}·Å^{-2}. In the selectivity maps, the selectivity is only computed if at least 200 product molecules have been formed; otherwise, it is shown in light grey. All heatmap plots are based on 225 (i.e., 15×15) KMC simulations at different (p_{CH_4}, p_{CO_2}) conditions.

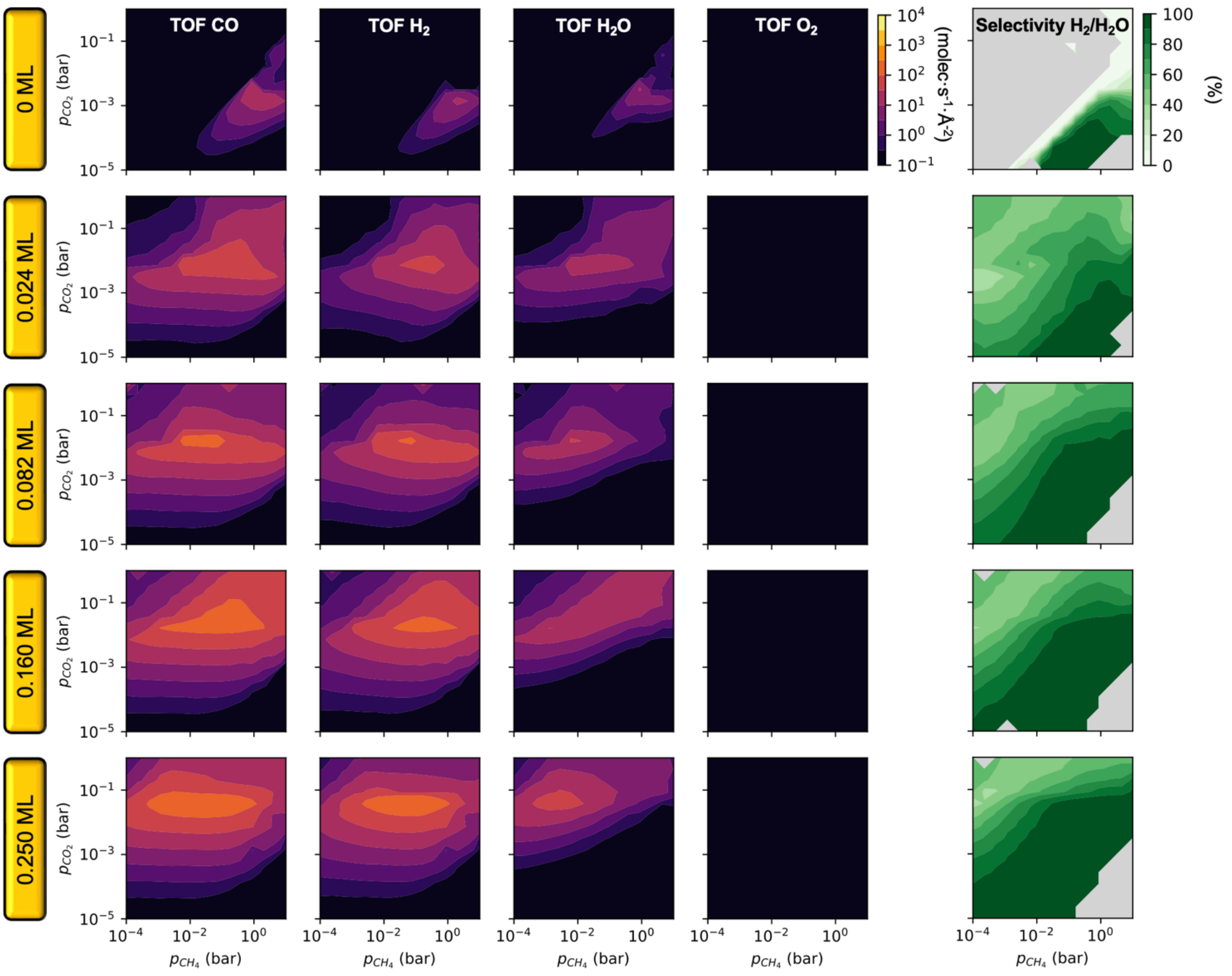

Figure S6. Computed TOF and H_2/H_2O selectivity for the DRM at 1000 K on Pt/HfC as a function of the Pt loading. In the TOF maps, black areas have a TOF < 10^{-1} molec·s^{-1}·$Å^{-2}$. In the selectivity maps, the selectivity is only computed if at least 200 product molecules have been formed; otherwise, it is shown in light grey. All heatmap plots are based on 225 (i.e., 15×15) KMC simulations at different (p_{CH_4}, p_{CO_2}) conditions.

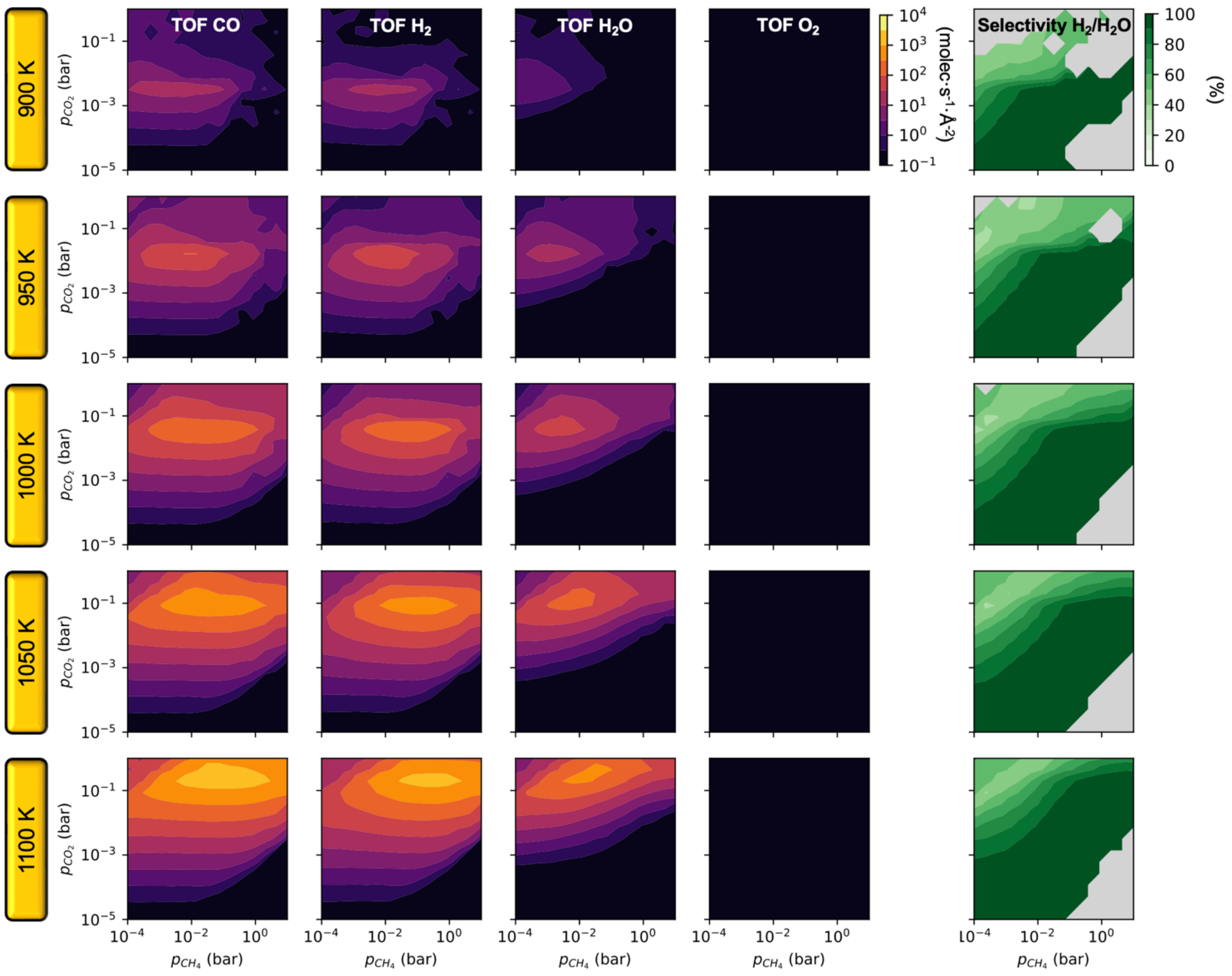

Figure S7. Computed TOF and H_2/H_2O selectivity for the DRM on Pt/HfC as a function of the reaction temperature in the range 900−1100 K. In the TOF maps, black areas have a TOF < 10^{-1} molec·s^{-1}·Å^{-2}. In the selectivity maps, the selectivity is only computed if at least 200 product molecules have been formed; otherwise, it is shown in light grey. All heatmap plots are based on 225 (i.e., 15×15) KMC simulations at different (p_{CH_4}, p_{CO_2}) conditions.

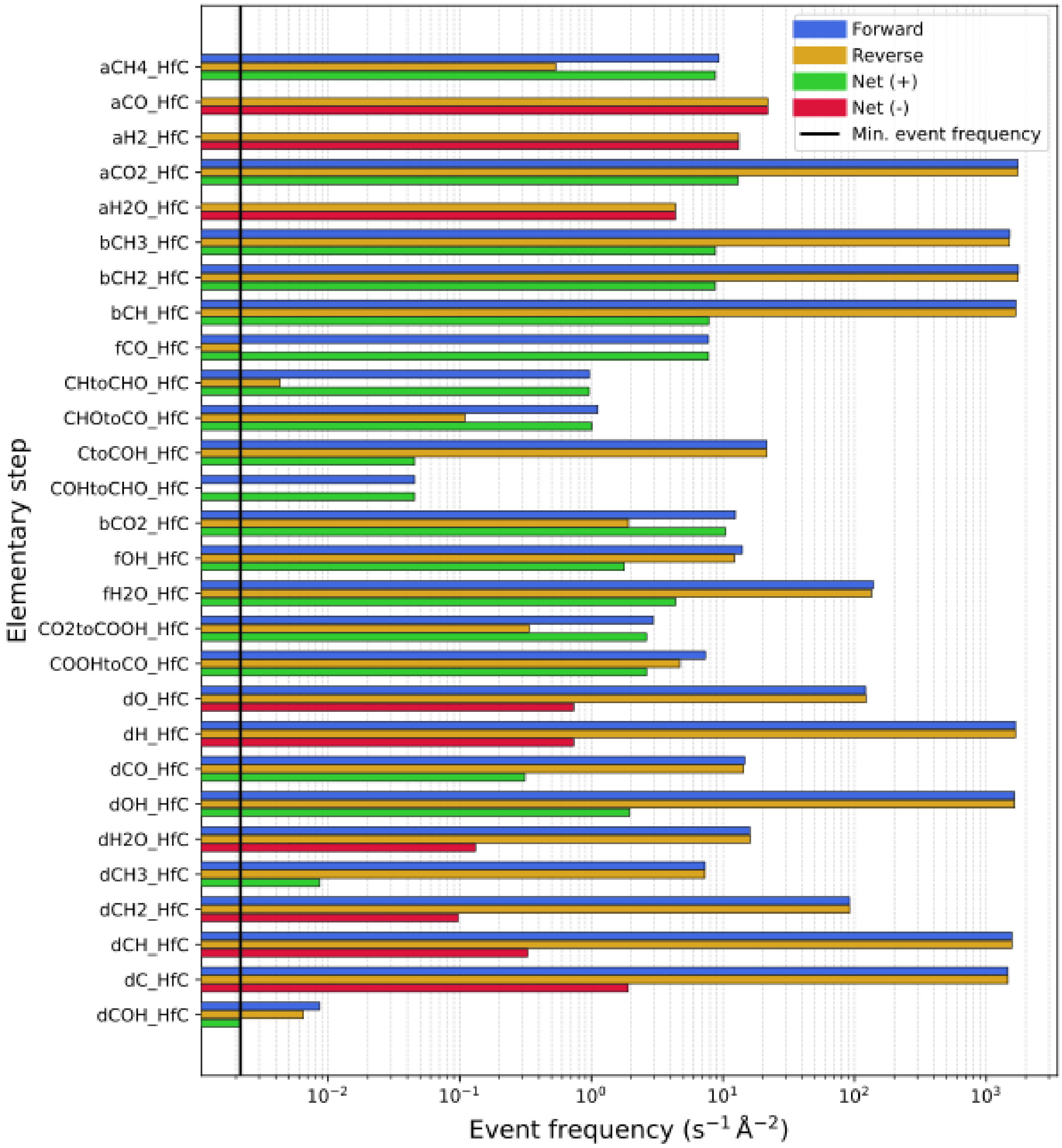

Figure S8. Process statistics for the DRM on HfC at 1000 K at the maximum of the H_2 production volcano (i.e., p_{CH_4} = 1.93·10^0 bar and p_{CO_2} = 1.39·10^{-3} bar, see Figure 3A). Note that the first 50% of the simulated time is assumed to correspond to the equilibration phase and therefore is not accounted for the process statistics.

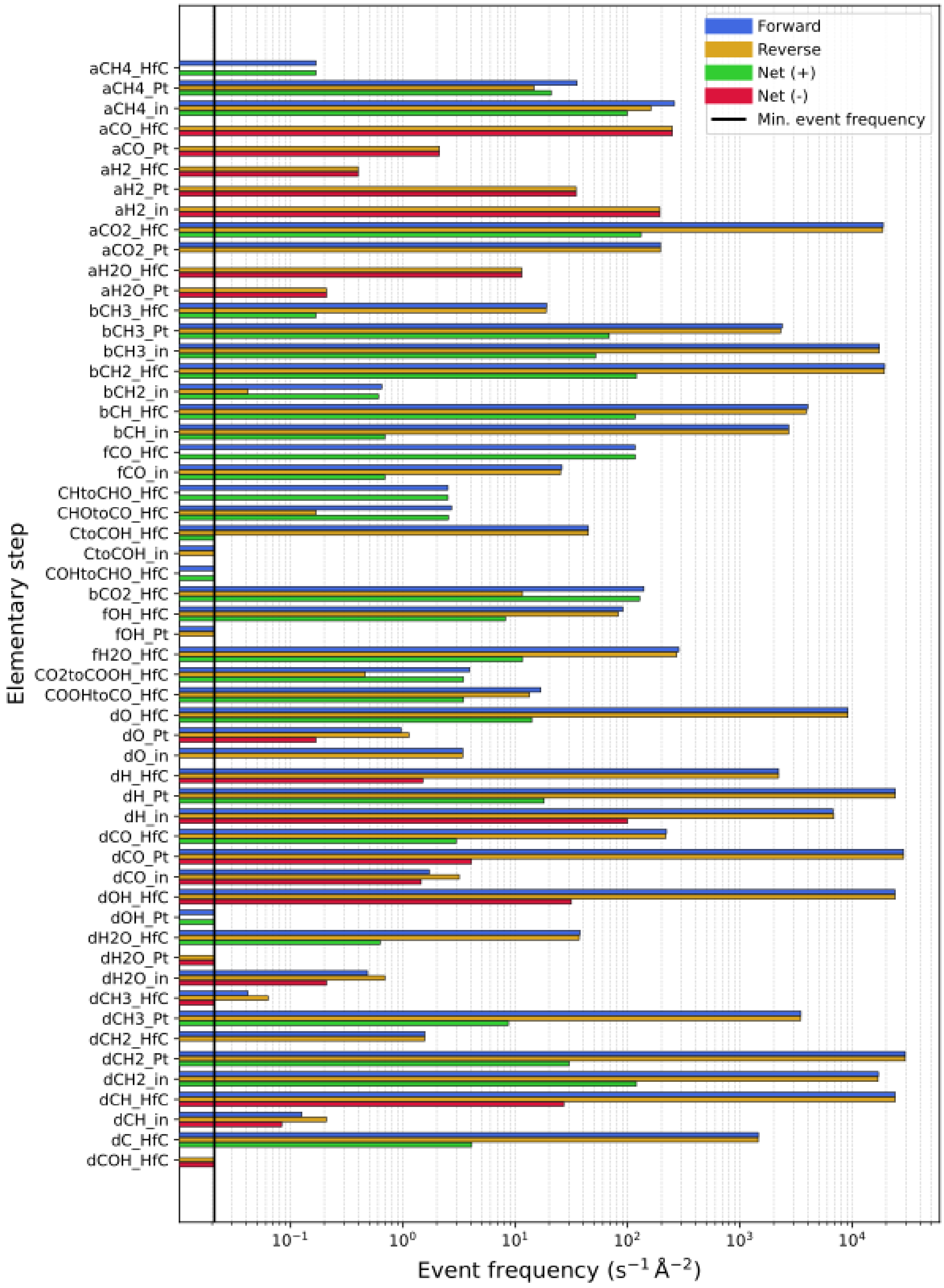

Figure S9. Process statistics for the DRM on Pt/HfC at 1000 K at the maximum of the H_2 production volcano (i.e., p_{CH_4} = 7.20·10^{-2} bar and p_{CO_2} = 3.73·10^{-2} bar, see Figure 3B). Note that the first 50% of the simulated time is assumed to correspond to the equilibration phase and therefore is not accounted for the process statistics.

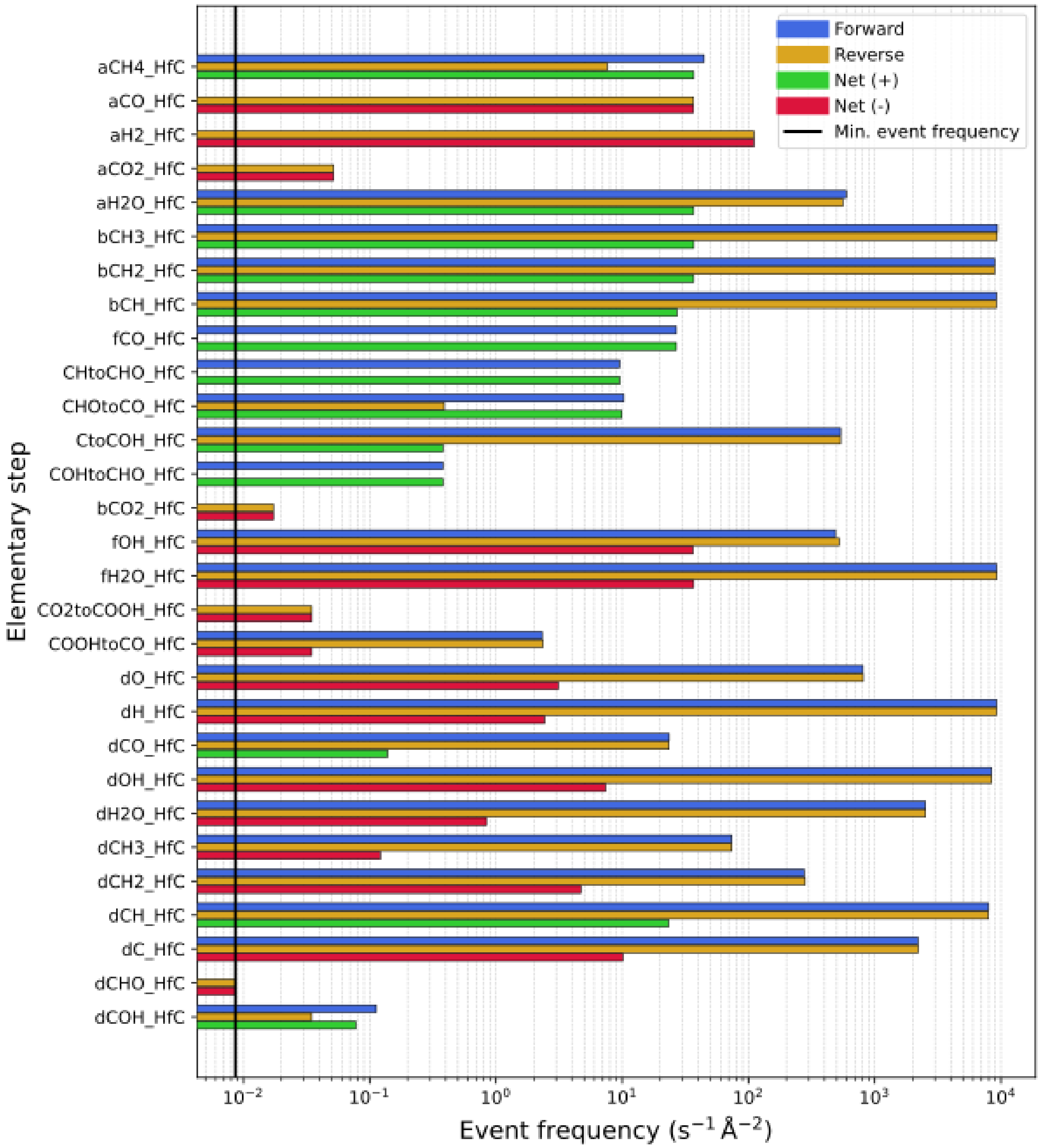

Figure S10. Process statistics for the SRM on HfC at 1000 K at the maximum of the H_2 production volcano (i.e., p_{CH_4} = $1.00 \cdot 10^{1}$ bar and p_{H_2O} = $6.11 \cdot 10^{-5}$ bar, see Figure S18A). Note that the first 50% of the simulated time is assumed to correspond to the equilibration phase and therefore is not accounted for the process statistics.

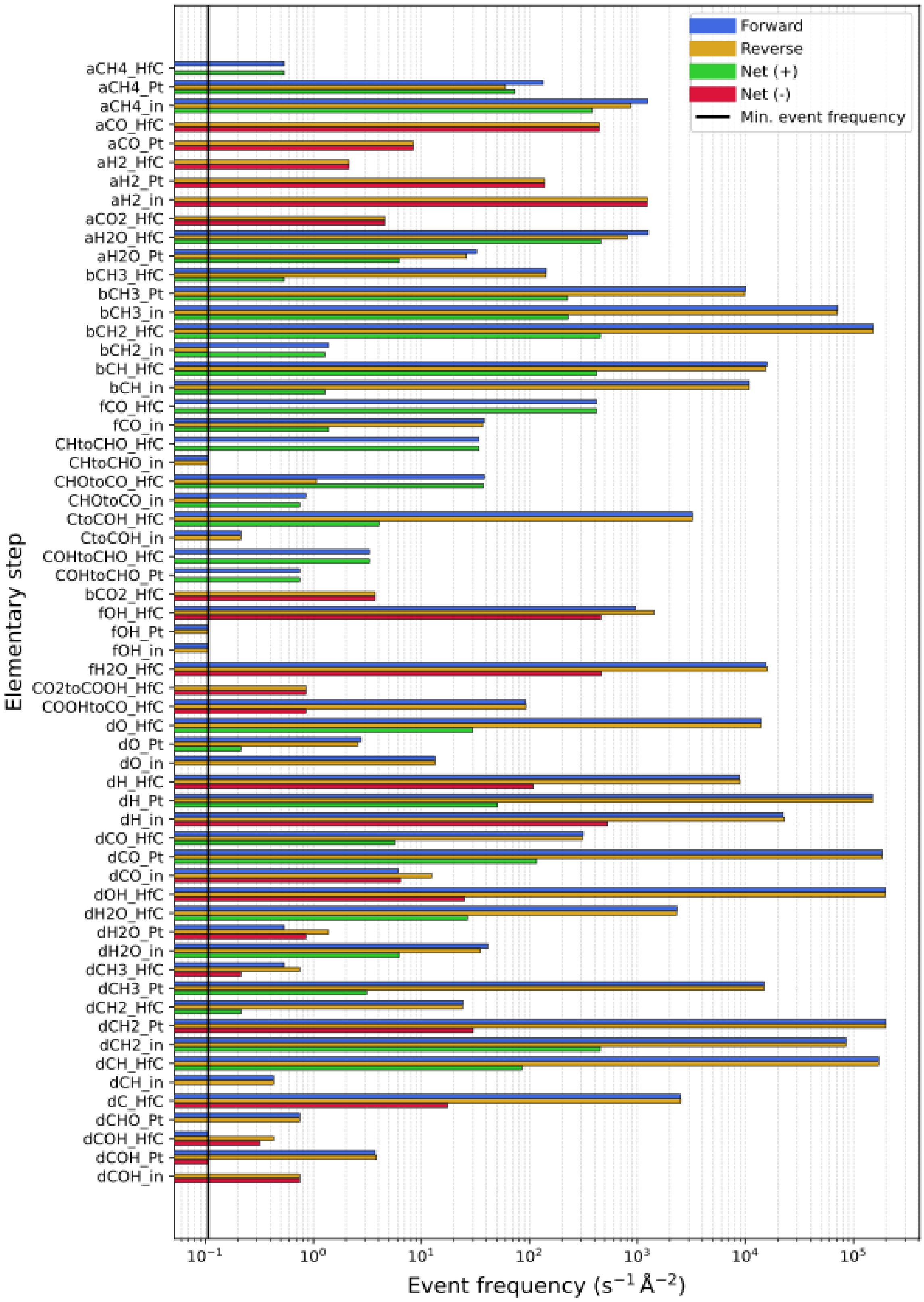

Figure S11. Process statistics for the SRM on Pt/HfC at 1000 K at the maximum of the H_2 production volcano (i.e., p_{CH_4} = 1.64·10^{-1} bar and p_{H_2O} = 1.39·10^{-4} bar, see Figure S18B). Note that the first 50% of the simulated time is assumed to correspond to the equilibration phase and therefore is not accounted for the process statistics.

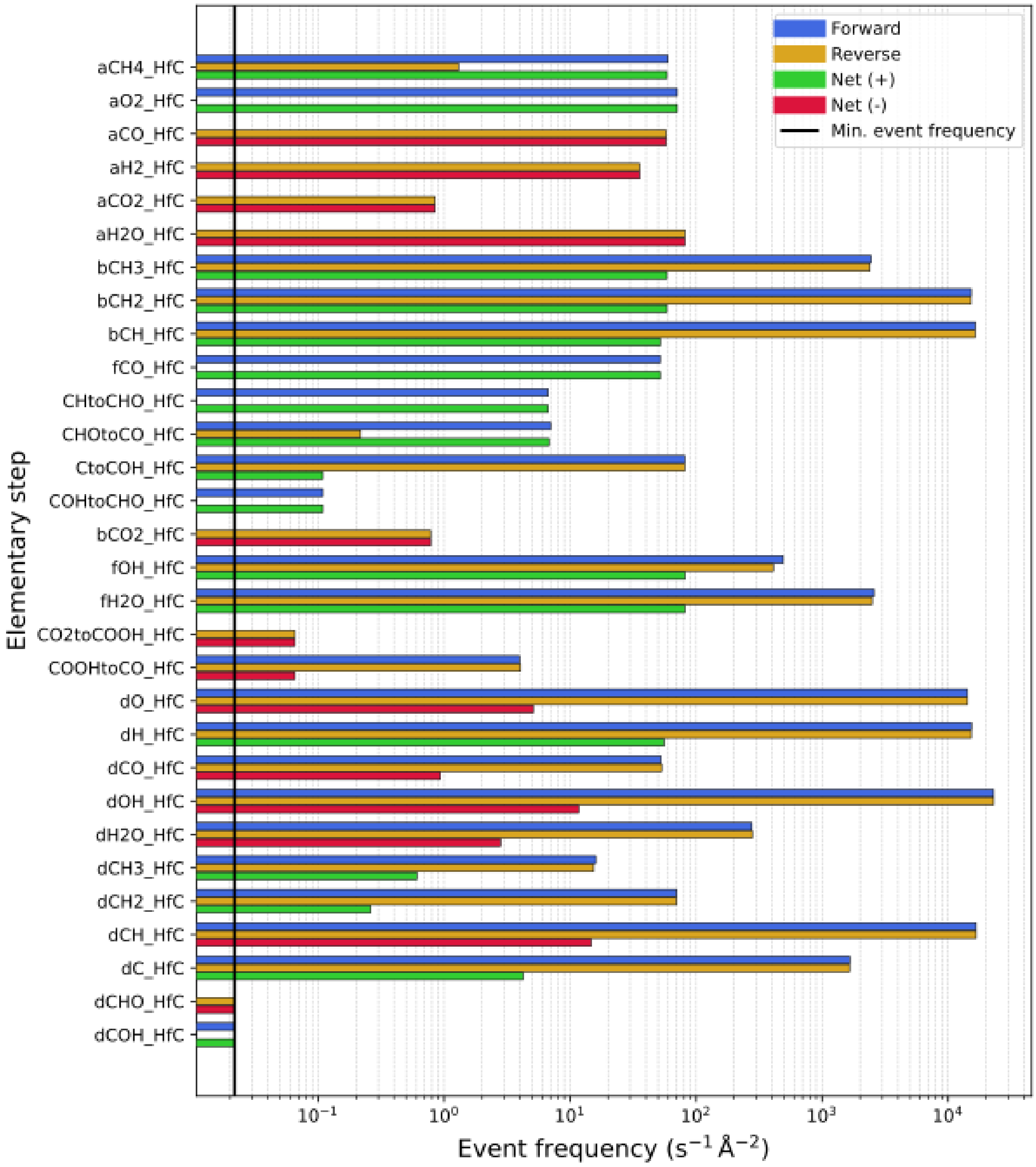

Figure S12. Process statistics for the POM on HfC at 1000 K at the maximum of the H_2 production volcano (i.e., p_{CH_4} = $1.00{\cdot}10^1$ bar and p_{O_2} = $3.16{\cdot}10^{-6}$ bar, see Figure S19A). Note that the first 50% of the simulated time is assumed to correspond to the equilibration phase and therefore is not accounted for the process statistics.

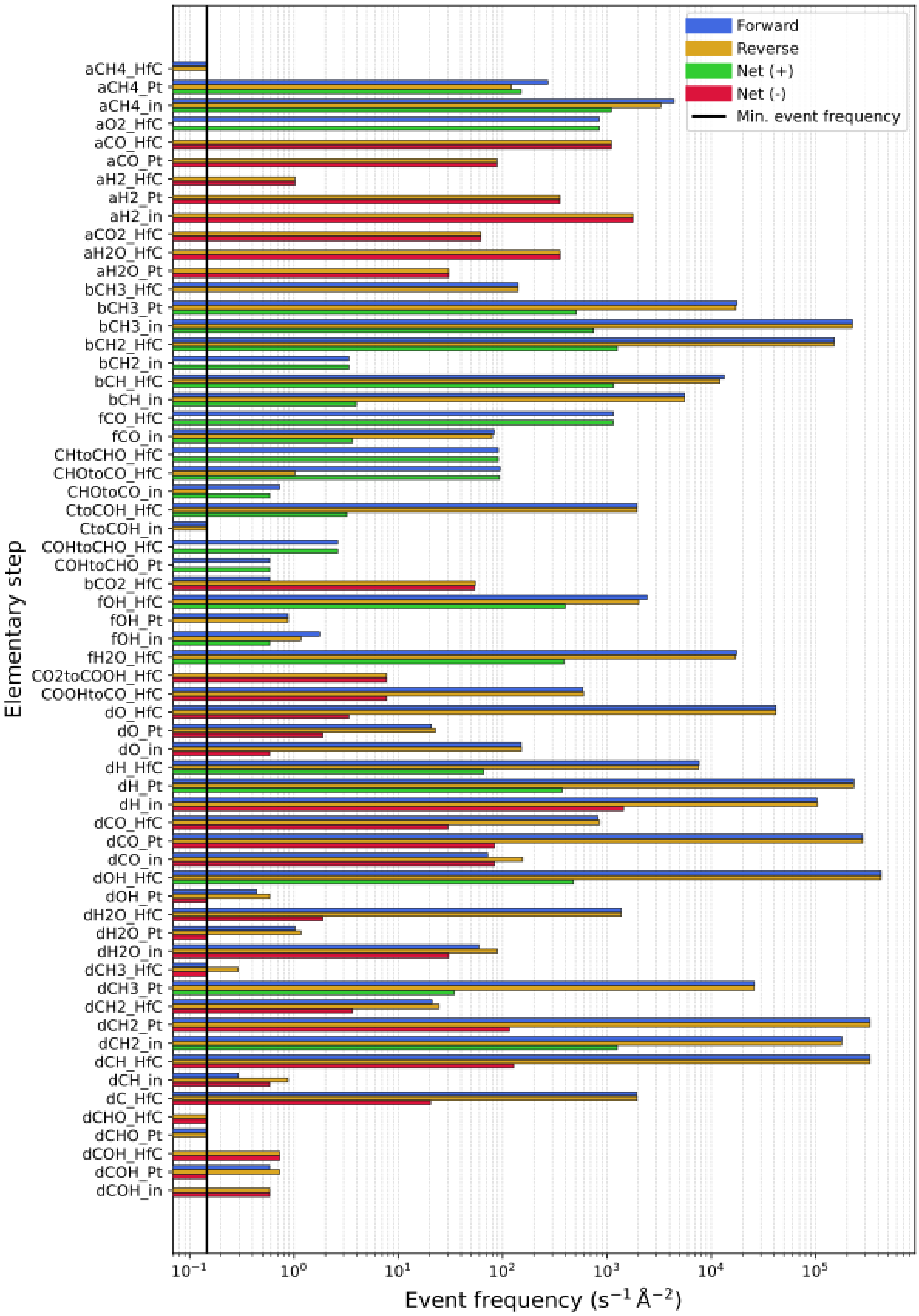

Figure S13. Process statistics for the POM on Pt/HfC at 1000 K at the maximum of the H_2 production volcano (i.e., p_{CH_4} = 1.64·10^{-1} bar and p_{O_2} = 8.48·10^{-5} bar, see Figure S19B). Note that the first 50% of the simulated time is assumed to correspond to the equilibration phase and therefore is not accounted for the process statistics.

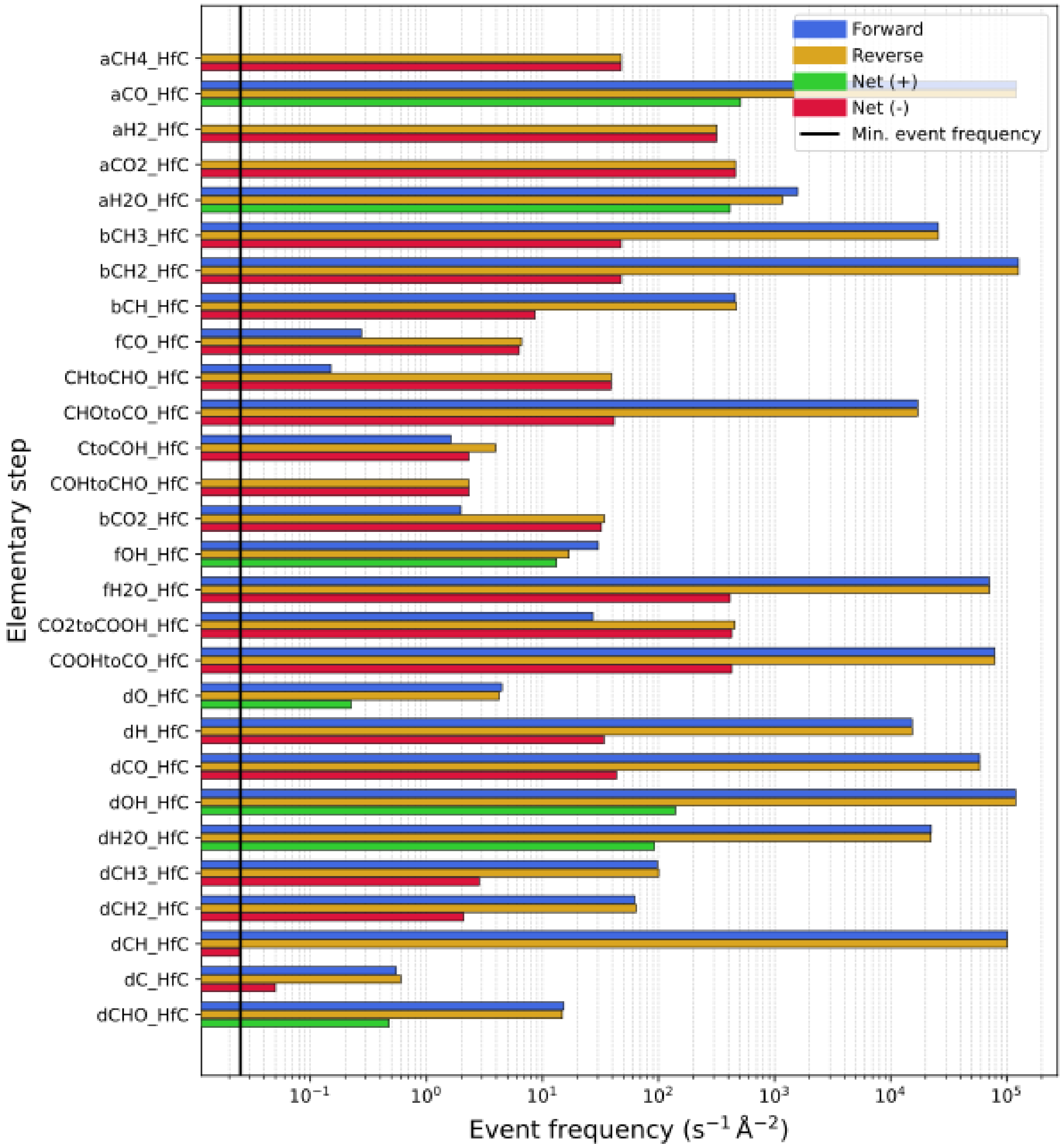

Figure S14. Process statistics for the WGS on HfC at 1000 K at the maximum of the H_2 production volcano (i.e., p_{CO} = $4.39 \cdot 10^0$ bar and p_{H_2O} = $1.64 \cdot 10^{-4}$ bar, see Figure S20A). Note that the first 50% of the simulated time is assumed to correspond to the equilibration phase and therefore is not accounted for the process statistics.

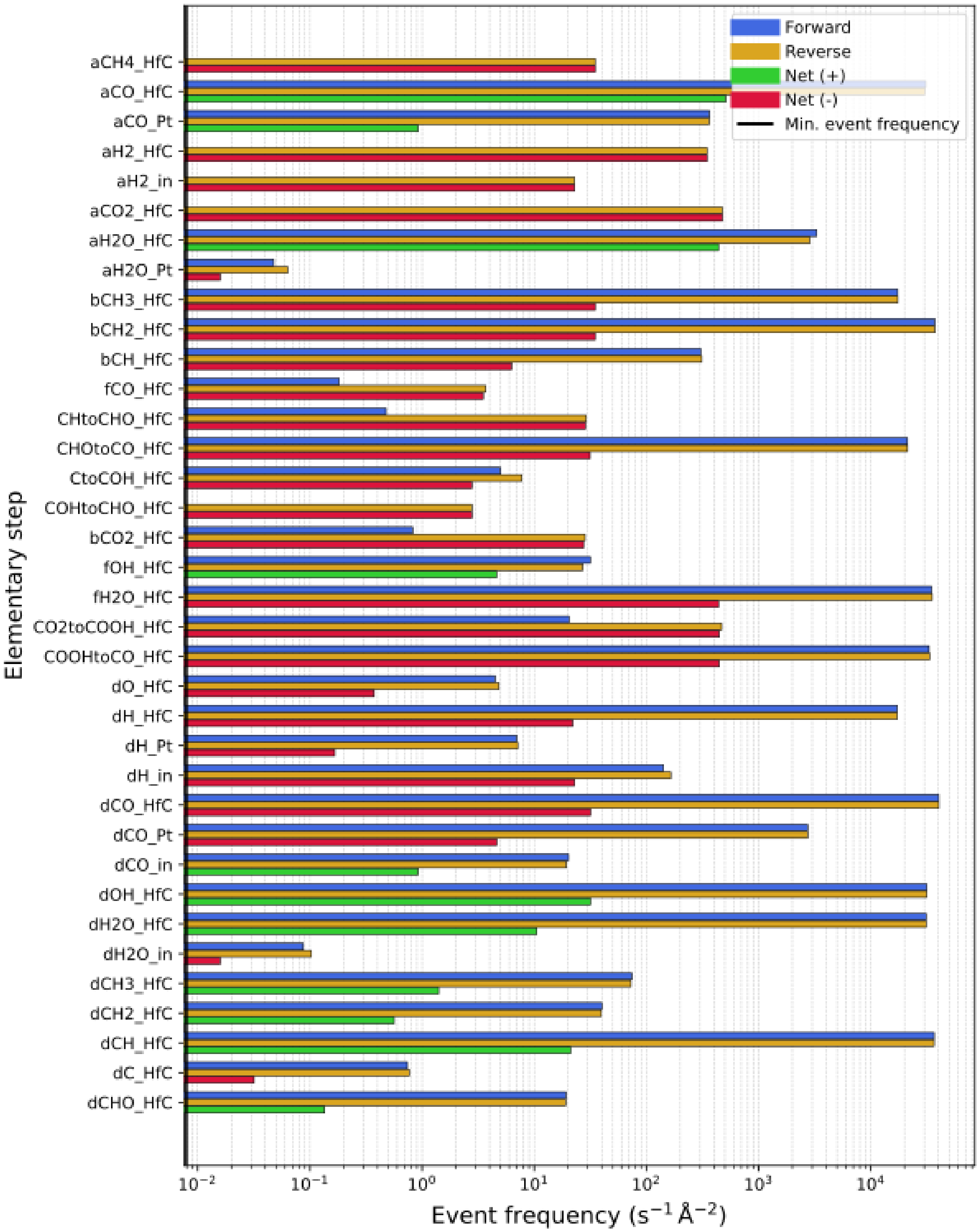

Figure S15. Process statistics for the WGS on Pt/HfC at 1000 K at the maximum of the H_2 production volcano (i.e., p_{CO} = 4.39·10^0 bar and p_{H_2O} = 3.73·10^{-4} bar, see Figure S20B). Note that the first 50% of the simulated time is assumed to correspond to the equilibration phase and therefore is not accounted for the process statistics.

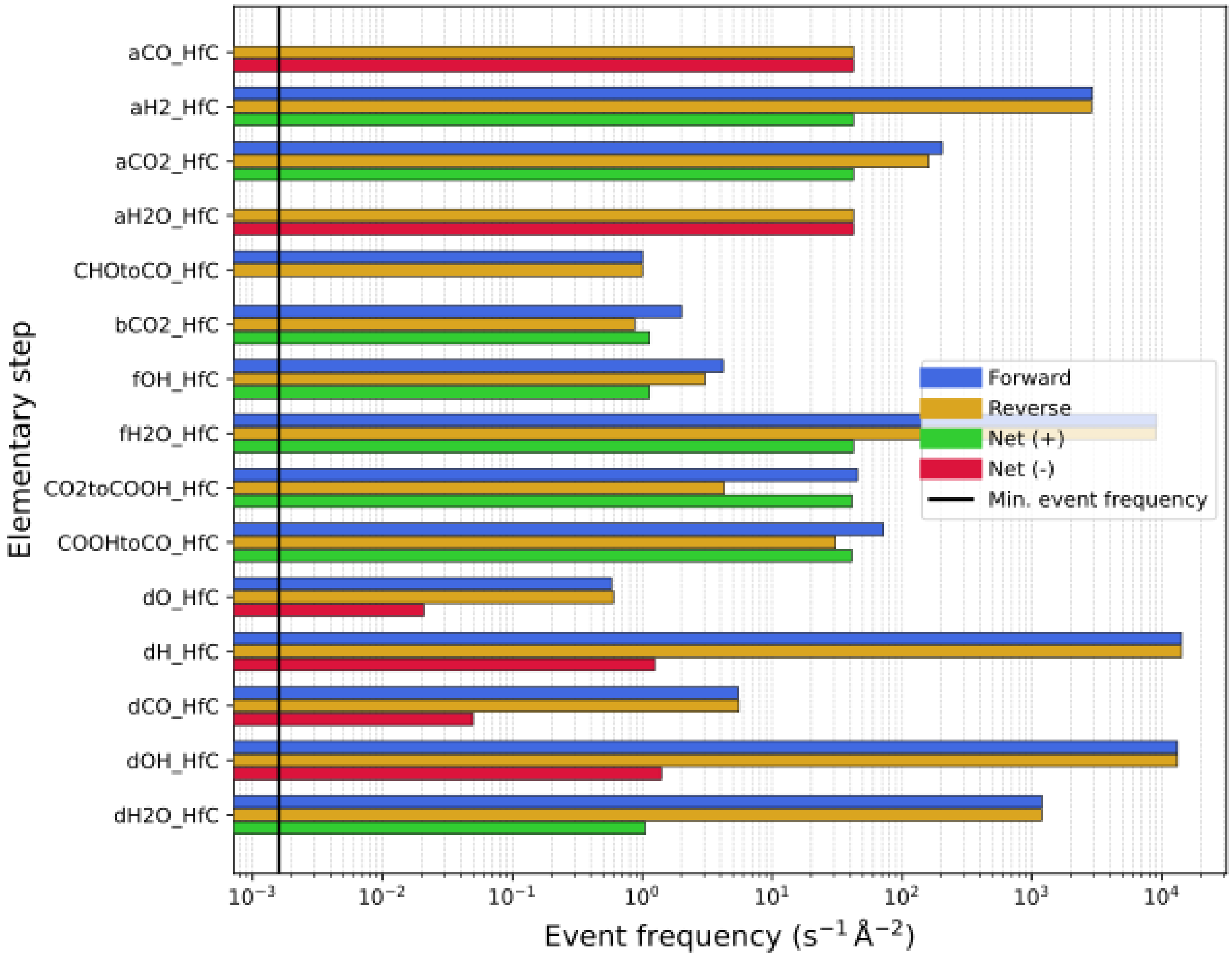

Figure S16. Process statistics for the RWGS on HfC at 1000 K at the maximum of the H_2 production volcano (i.e., $p_{CO_2} = 1.18 \cdot 10^{-4}$ bar and $p_{H_2} = 1.00 \cdot 10^{1}$ bar, see Figure S21A). Note that the first 50% of the simulated time is assumed to correspond to the equilibration phase and therefore is not accounted for the process statistics.

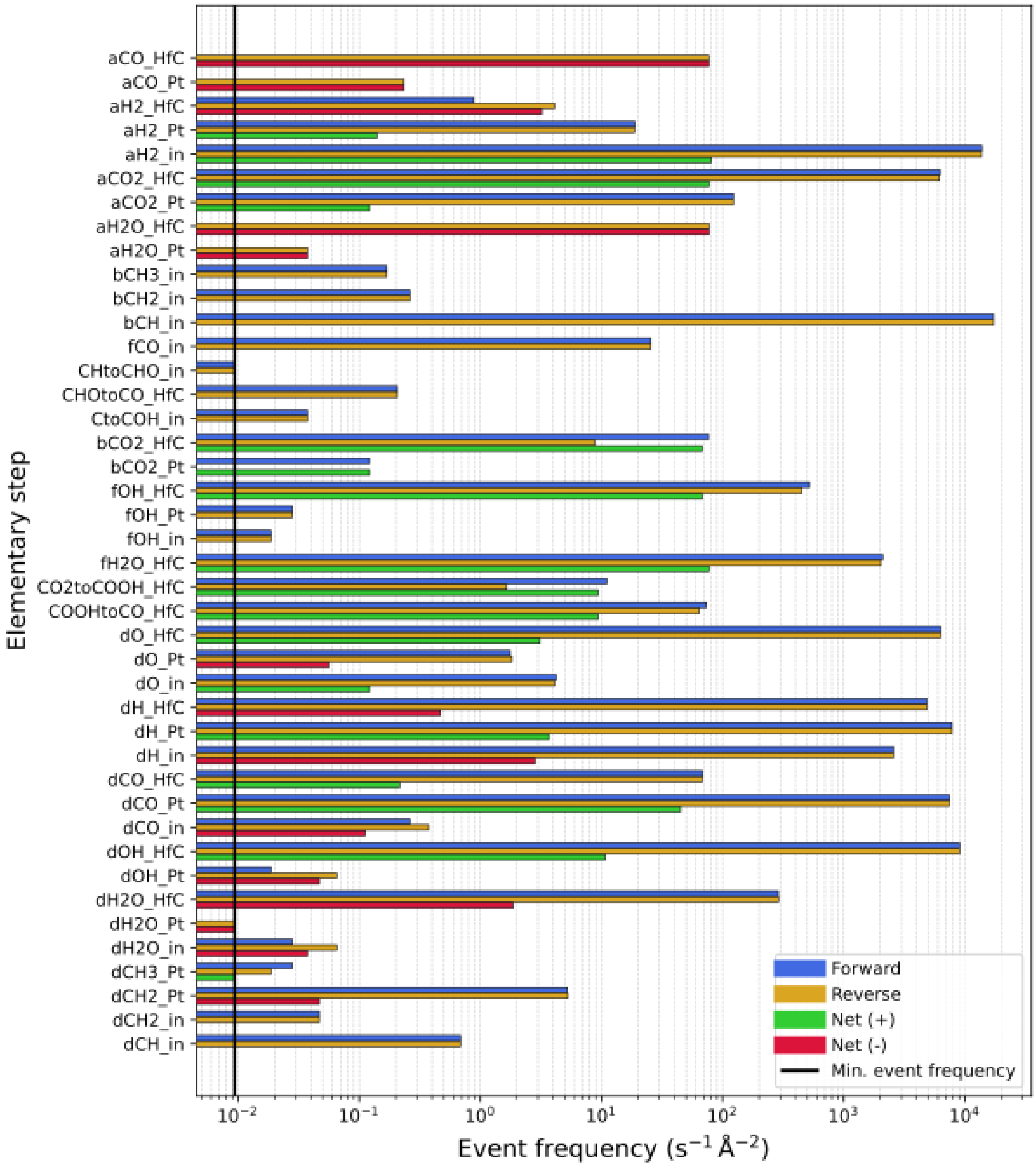

Figure S17. Process statistics for the RWGS on Pt/HfC at 1000 K at the maximum of the H_2 production volcano (i.e., $p_{CO_2} = 1.64 \cdot 10^{-2}$ bar and $p_{H_2} = 2.28 \cdot 10^{-4}$ bar, see Figure S21B). Note that the first 50% of the simulated time is assumed to correspond to the equilibration phase and therefore is not accounted for the process statistics.

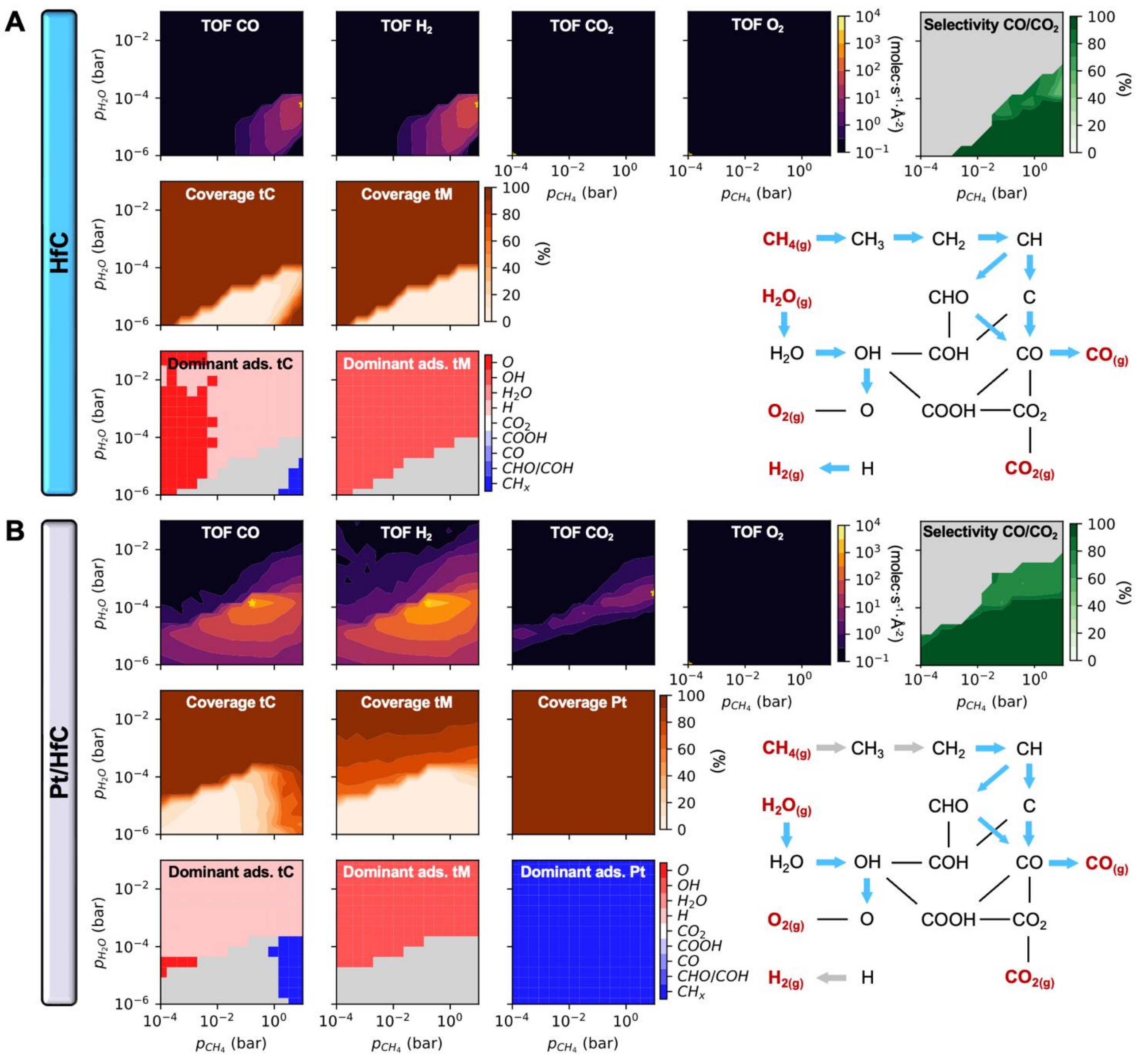

Figure S18. Computed TOF, CO/CO$_2$ selectivity, total surface coverage, kinetic phase diagrams and dominant reaction pathway for the SRM at 1000 K on **(A)** HfC and **(B)** Pt/HfC. In the TOF maps, black areas have a TOF < 10^{-1} molec·s^{-1}·Å^{-2}. In the selectivity maps, the selectivity is only computed if at least 200 product molecules have been formed; otherwise, it is shown in light grey. In the kinetic phase diagrams, non-grey areas correspond to regions where the overall coverage is higher than 50%, and the colour indicates the most abundant species at those conditions. The dominant reaction pathways correspond to the highest H_2 TOF (golden '*' marker in TOF maps) and are obtained from the process statistics plots in Figure S10 (HfC) and Figure S11 (Pt/HfC). Blue (HfC) and grey (Pt/HfC) arrows indicate which region is active for that step, and the arrow thickness is proportional to the event frequency. All heatmap plots are based on 225 (i.e., 15×15) KMC simulations at different (p_{CH_4}, p_{H_2O}) conditions.

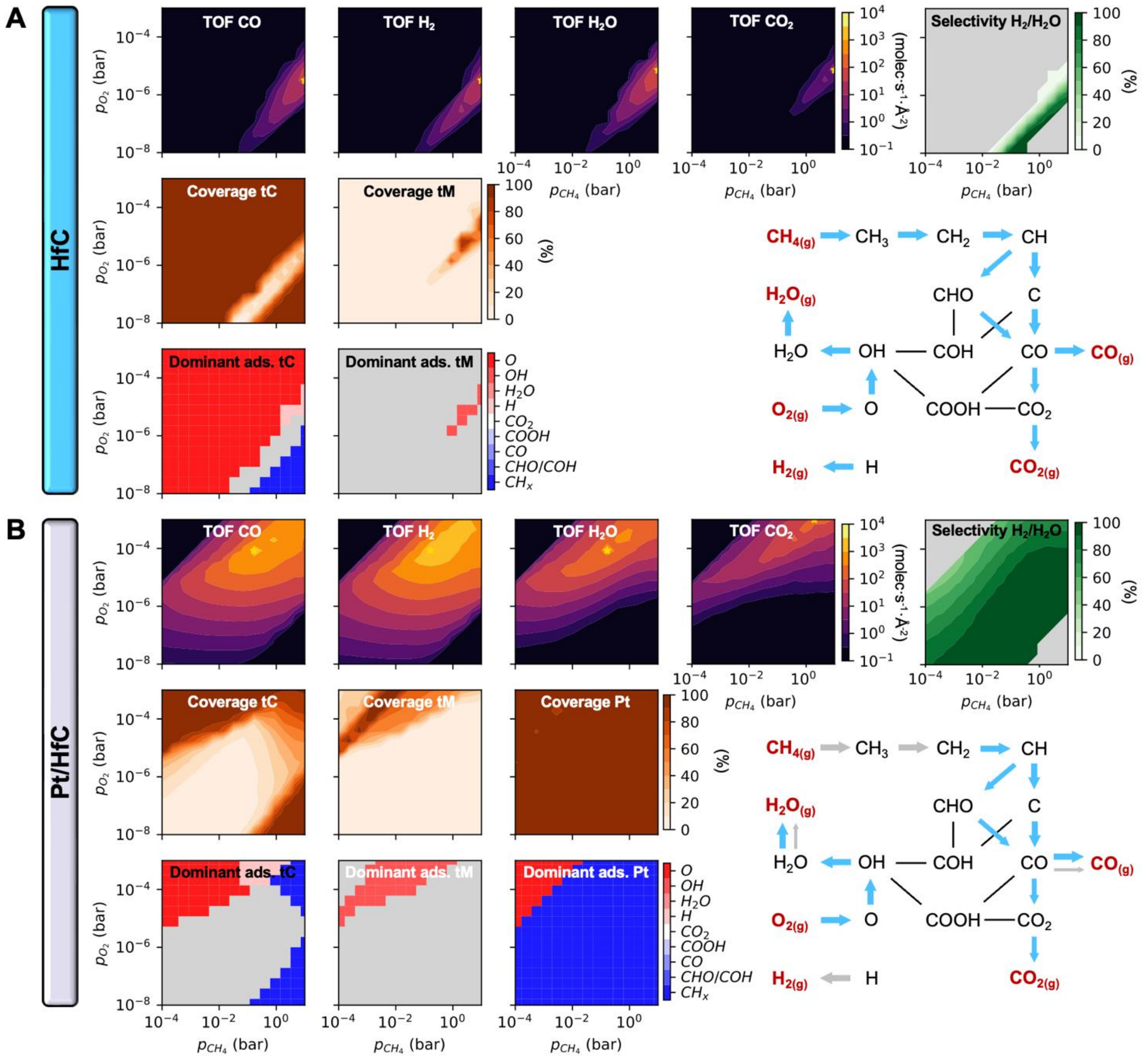

Figure S19. Computed TOF, H_2/H_2O selectivity, total surface coverage, kinetic phase diagrams and dominant reaction pathway for the POM at 1000 K on **(A)** HfC and **(B)** Pt/HfC. In the TOF maps, black areas have a TOF < 10^{-1} molec·s^{-1}·Å^{-2}. In the selectivity maps, the selectivity is only computed if at least 200 product molecules have been formed; otherwise, it is shown in light grey. In the kinetic phase diagrams, non-grey areas correspond to regions where the overall coverage is higher than 50%, and the colour indicates the most abundant species at those conditions. The dominant reaction pathways correspond to the highest H_2 TOF (golden '*' marker in TOF maps) and are obtained from the process statistics plots in Figure S12 (HfC) and Figure S13 (Pt/HfC). Blue (HfC) and grey (Pt/HfC) arrows indicate which region is active for that step, and the arrow thickness is proportional to the event frequency. All heatmap plots are based on 225 (i.e., 15×15) KMC simulations at different (p_{CH_4}, p_{O_2}) conditions.

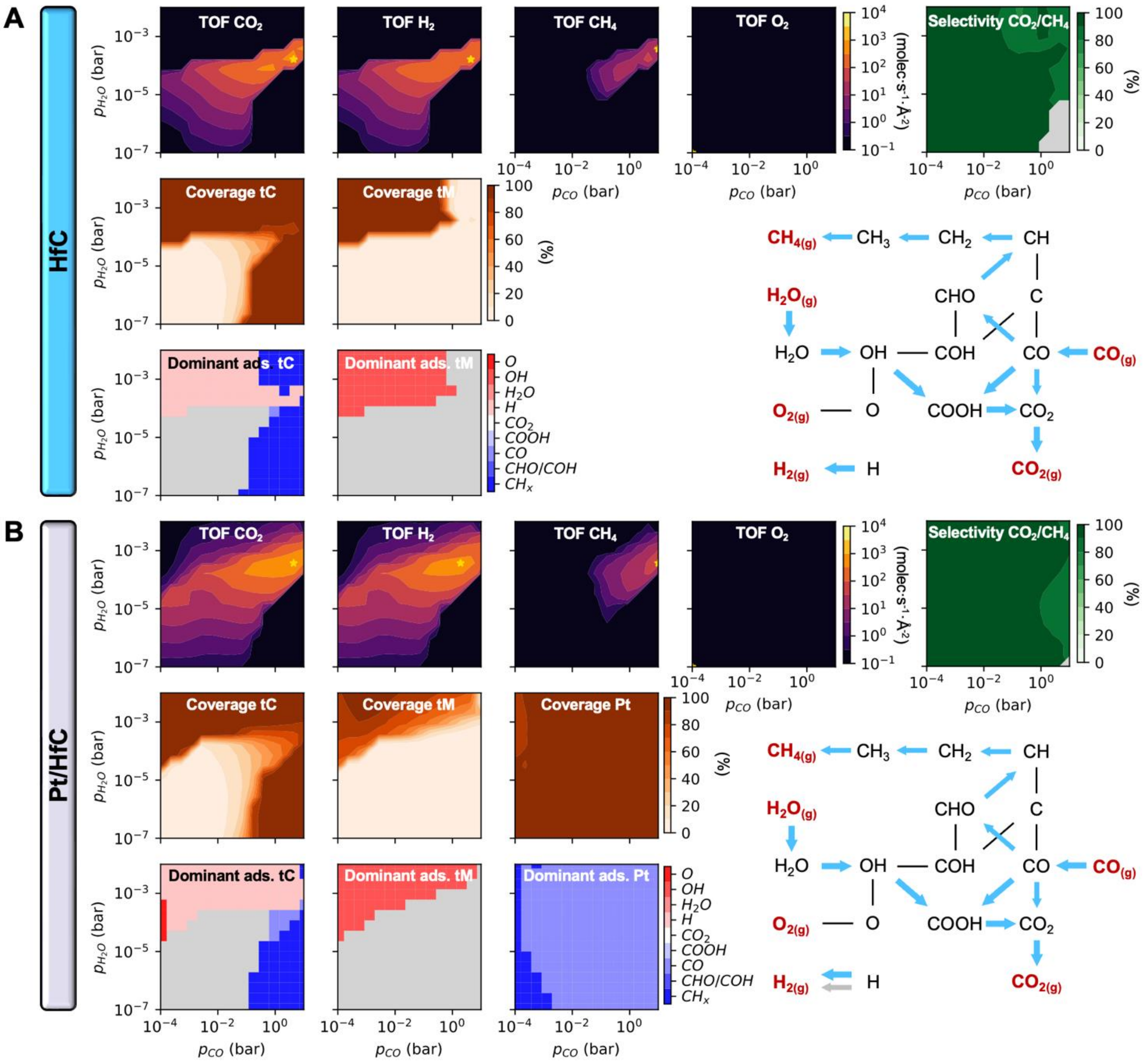

Figure S20. Computed TOF, CO_2/CH_4 selectivity, total surface coverage, kinetic phase diagrams and dominant reaction pathway for the WGS reaction at 1000 K on **(A)** HfC and **(B)** Pt/HfC. In the TOF maps, black areas have a TOF < 10^{-1} molec·s^{-1}·Å^{-2}. In the selectivity maps, the selectivity is only computed if at least 200 product molecules have been formed; otherwise, it is shown in light grey. In the kinetic phase diagrams, non-grey areas correspond to regions where the overall coverage is higher than 50%, and the colour indicates the most abundant species at those conditions. The dominant reaction pathways correspond to the highest H_2 TOF (golden '*' marker in TOF maps) and are obtained from the process statistics plots in Figure S14 (HfC) and Figure S15 (Pt/HfC). Blue (HfC) and grey (Pt/HfC) arrows indicate which region is active for that step, and the arrow thickness is proportional to the event frequency. All heatmap plots are based on 225 (i.e., 15×15) KMC simulations at different (p_{CO}, p_{H_2O}) conditions.

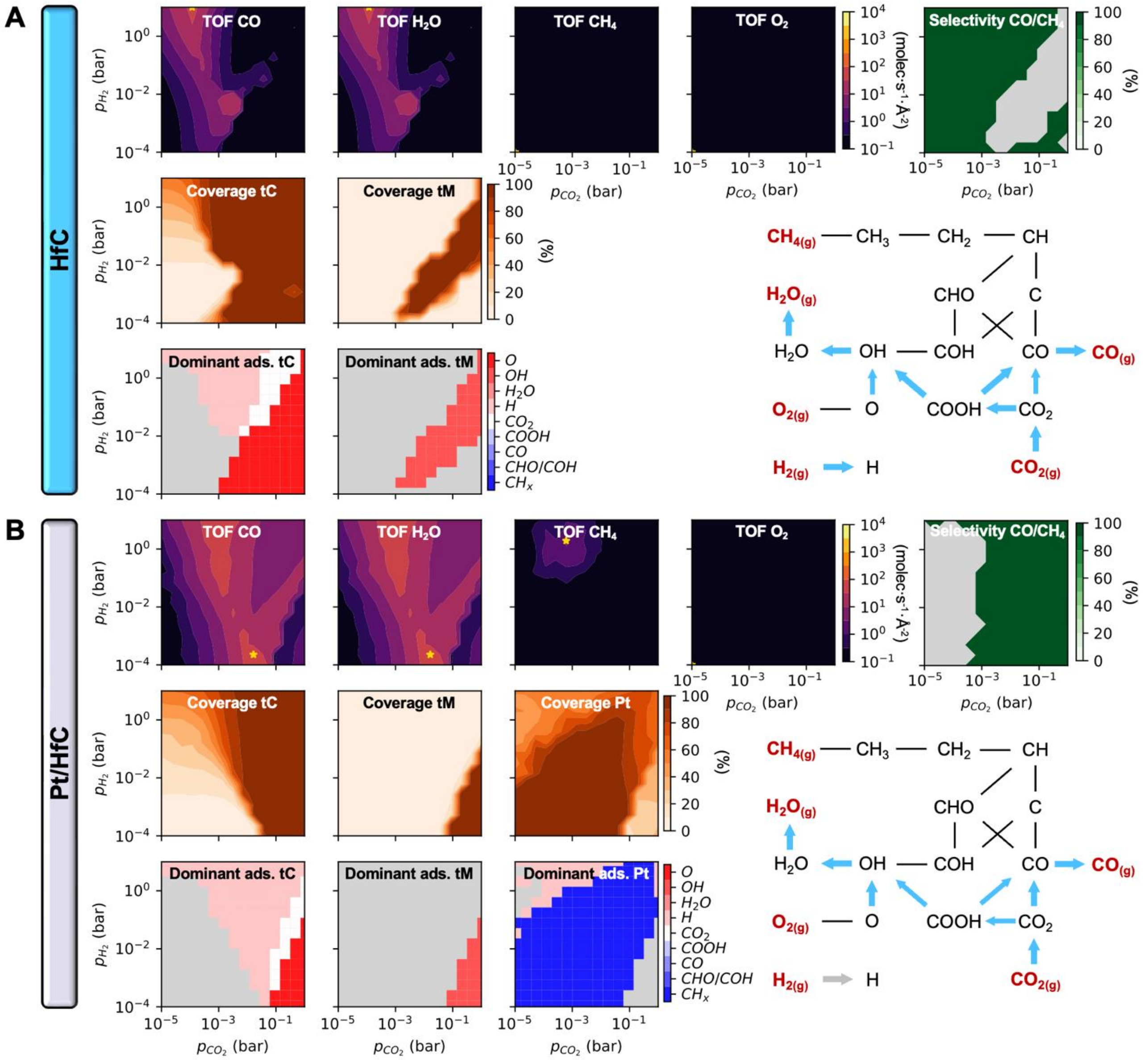

Figure S21. Computed TOF, CO/CH$_4$ selectivity, total surface coverage, kinetic phase diagrams and dominant reaction pathway for the RWGS reaction at 1000 K on **(A)** HfC and **(B)** Pt/HfC. In the TOF maps, black areas have a TOF < 10^{-1} molec·s^{-1}·Å^{-2}. In the selectivity maps, the selectivity only computed if at least 200 product molecules have been formed; otherwise, it is shown in light grey. In the kinetic phase diagrams, non-grey areas correspond to regions where the overall coverage is higher than 50%, and the colour indicates the most abundant species at those conditions. The dominant reaction pathways correspond to the highest CO TOF (golden '*' marker in TOF maps) and are obtained from the process statistics plots in Figure S16 (HfC) and Figure S17 (Pt/HfC). Blue (HfC) and grey (Pt/HfC) arrows indicate which region is active for that step, and the arrow thickness is proportional to the event frequency. All heatmap plots are based on 225 (i.e., 15×15) KMC simulations at different (p_{CO_2}, p_{H_2}) conditions.

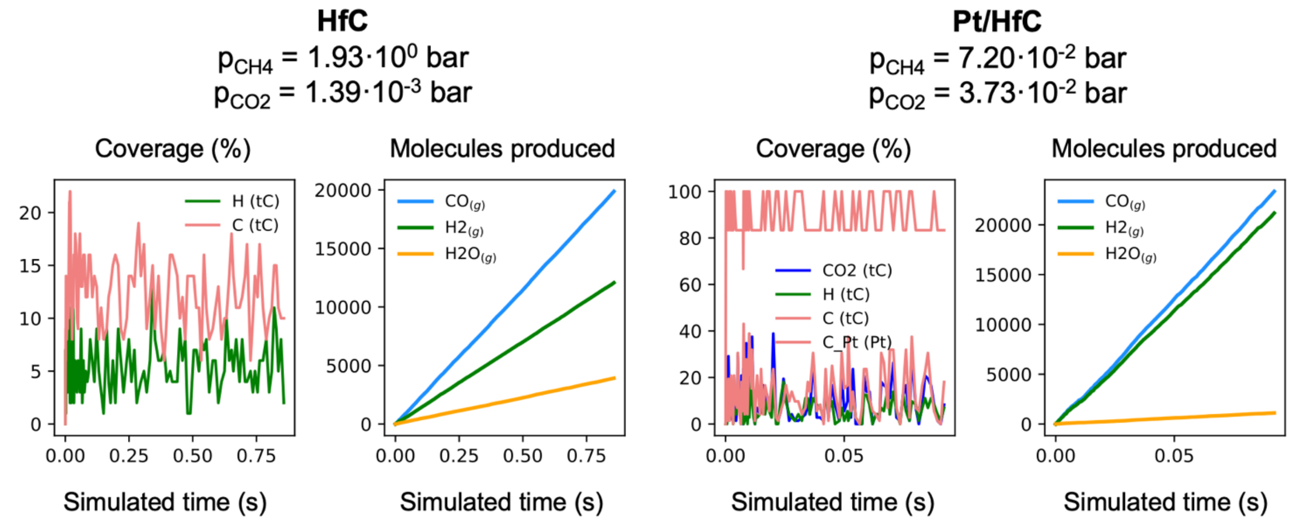

Figure S22. Surface coverage (normalised per site type) and product molecules formed as a function of the simulation time for the DRM at 1000 K on HfC (left) and Pt/HfC (right) at the (p_{CH_4}, p_{CO_2}) conditions corresponding to the highest H_2 TOF. Adsorbates with an average coverage lower than 5% are not included in the coverage plots.

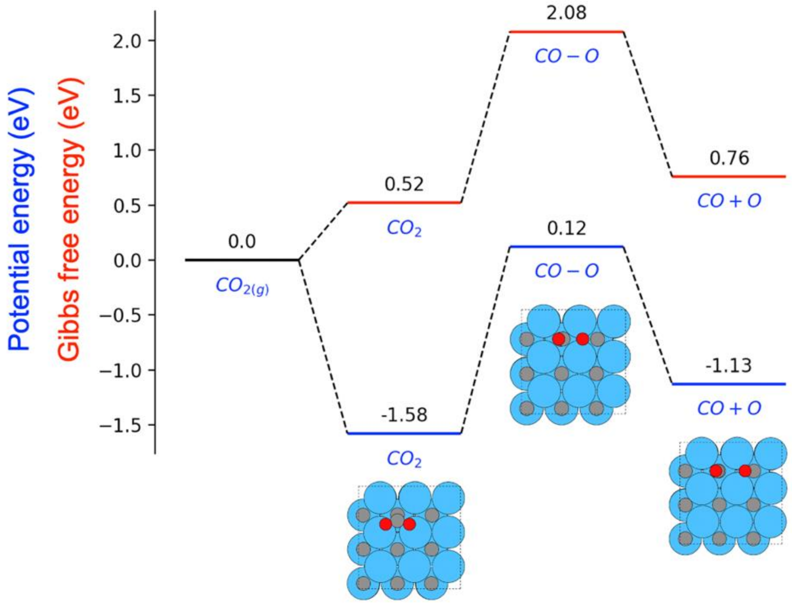

Figure S23. Potential energy (blue) and Gibbs free energy (red) diagrams for CO_2 adsorption and dissociation on clean HfC. The Gibbs free energy has been calculated at 1000 K and $p_{CO2} = 3.72 \cdot 10^{-2}$ bar by means of the ASE thermochemistry module[1], using the ideal gas model for gas-phase molecules and the harmonic oscillator model for adsorbed species.

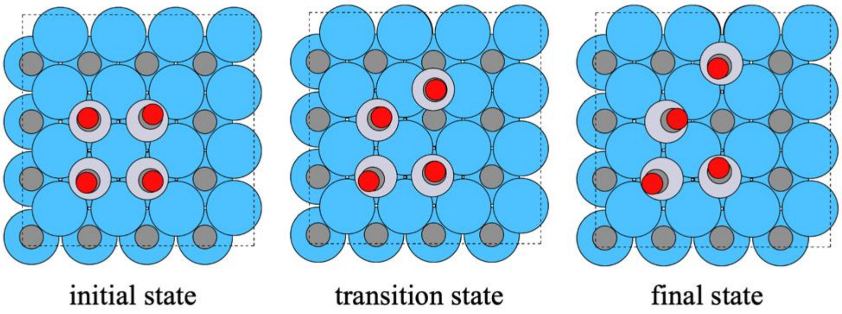

Figure S24. Top views for the initial, transition, and final states for CO-covered Pt_4 fragmentation to Pt_3+Pt.

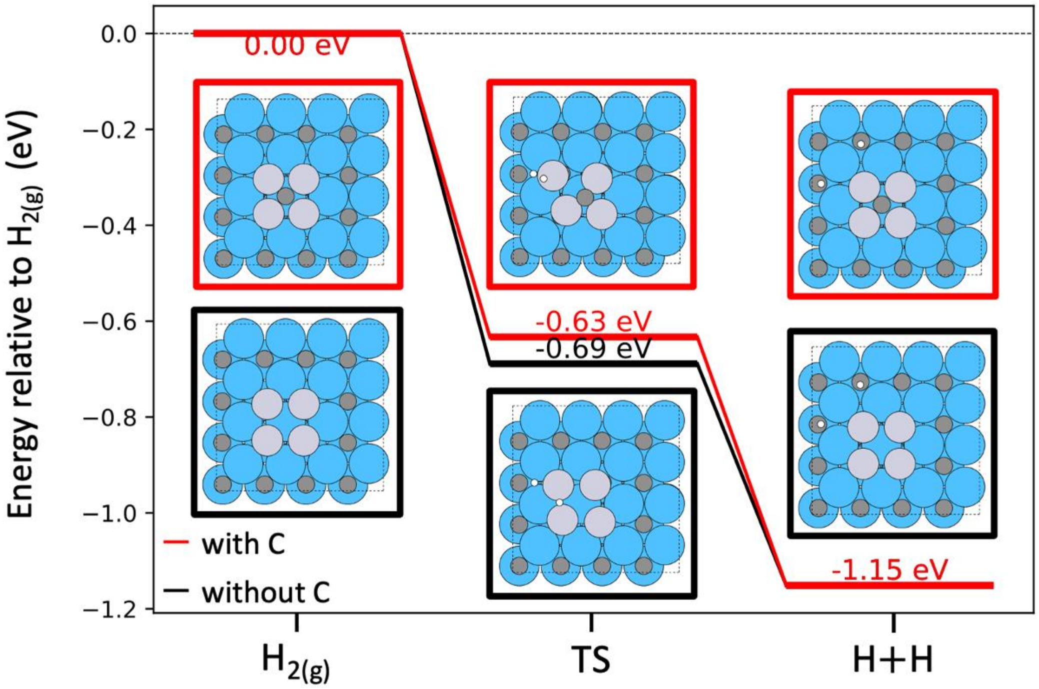

Figure S25. Potential energies for the H_2 dissociation transition state and the H+H state relative to $H_{2(g)}$ on Pt_4/HfC with (red) and without (black) a C atom adsorbed on the Pt_4 cluster hollow site.

Supporting Notes

Note S1. Calculation of rate constants

In *Zacros*, the user must supply the pre-exponential factor (A) and the energy barrier ($\Delta E^{\neq}$) for each elementary step. Then, *Zacros* calculates the corresponding rate constant for each step as follows:

$$k = A \cdot exp\left(-\frac{\Delta E^{\neq}}{k_B T}\right) \quad (1)$$

The entropic contributions are inherently included in the calculation of the pre-exponential factors through the partition functions of reactants and transition states.

In our work, all pre-exponential factors were computed from first-principles calculations based on Transition State Theory (TST), as described in Refs. 2 and [3]. In summary, for gas-phase species, we used the ideal gas approximation, which considers the translational, rotational, and vibrational components of the partition function. For adsorbed species, we used the harmonic oscillator model, which assumes that the partition function of the adsorbed species only vibrational components, as they have limited translational and rotational freedom on the surface. The vibrational frequencies of all species were also obtained from DFT calculations.

Note S2. Modelling the effect of lateral interactions using the BEP relations

In *Zacros*, the forward ($E_{fwd}(\sigma)$) and reverse ($E_{rev}(\sigma)$) energy barriers for a given step at configuration σ are parameterised in terms of a Brønsted-Evans-Polanyi (BEP) relationship as follows:

$$E_{fwd}(\sigma) = max\left[0, \Delta \mathrm{E}_{rxn}(\sigma), E_{fwd,0} + \omega\left(\Delta \mathrm{E}_{rxn}(\sigma) - \Delta \mathrm{E}_{rxn,0}\right)\right] \quad (2)$$

$$E_{rev}(\sigma) = max\left[-\Delta \mathrm{E}_{rxn}(\sigma), 0, E_{rev,0} - (1-\omega)\left(\Delta \mathrm{E}_{rxn}(\sigma) - \Delta \mathrm{E}_{rxn,0}\right)\right] \quad (3)$$

where $\Delta \mathrm{E}_{rxn}(\sigma)$ and $\Delta \mathrm{E}_{rxn,0}$ are the reaction energies at configuration σ and at zero-coverage limit, respectively (calculated by the cluster-expansion); $E_{fwd,0}$ and $E_{rev,0}$ are the forward and reverse energy barriers at zero-coverage limit, respectively (calculated from DFT); ω is the so-called proximity factor ranging from 0.0 for an initial-state-like transition state to 1.0 for a final-state-like transition state, and the max operator filters negative values (as well as values less than $\Delta \mathrm{E}_{rxn}(\sigma)$ if the latter is positive). We have taken ω as 0.0 for non-activated adsorptions steps (i.e., initial-state-like transition state) and 0.5 for the rest.

Note S3. Ensuring that the steady-state is reached in all KMC simulations

There are several approaches to determining whether a KMC simulation has reached the steady-state or not. In practice, one can manually analyse if the coverage of adsorbed species and the TOF for each product at different time windows fluctuates around a constant value. If the simulation has not reached steady-state, one can analyse the event frequencies of all elementary steps, and carefully downscale manually the rate constants of the very fast and equilibrated steps to access longer time scales in which the system reaches steady state. In this work, however, this approach is not practical, as we are dealing with thousands of simulations at different operating conditions, which would require different downscaling factors, and a complex reaction network involving numerous adsorbed and gas-phase species.

Therefore, we have implemented the following strategy to ensure that all simulations reach steady-state:

1) **Dynamic scaling of the rate constants of fast and equilibrated events:** In KMC simulations, some processes might have rates that are much higher than those of all the other processes, resulting in these fast processes quickly reaching quasi-equilibrium. Yet, most of the computational time will be wasted only simulating these extremely frequent processes. To overcome this issue and accelerate the simulations, we have enabled dynamic detection of fast-processes and scaling of the kinetic constants on-the-fly. This procedure, named "stiffness scaling", is implemented in *Zacros* along the lines of previously published algorithms[4,5,6]. Specifically, we have used the *prats2024* stiffness scaling algorithm with the following parameters:
    - check_every 500000
    - min_separation 50.0
    - max_separation 100.0
    - tol_part_equil_ratio 0.05
    - upscaling_factor 5.0
    - upscaling_limit 100.0
    - downscaling_limit 2.0
    - min_noccur 10
2) **Automatic steady-state detection:** We have developed and implemented a new functionality in the Python library *ZacrosTools* (https://github.com/hprats/ZacrosTools) which can automatically detect whether a simulation has likely reached steady state or not. This functionality applies the following criteria:
    - Positive or negative trends in the lattice energy vs. simulated time. The lattice energy is a function of the coverage of each possible adsorbed species. If the number of all adsorbate species as a function of simulated time fluctuates around a constant value, the lattice energy will also fluctuate around a constant value. A positive or negative trend in the lattice energy suggests that the system has not reached the steady state.

- Non-linearity in the simulated time vs. number of KMC steps. The time increment in Zacros corresponds to the minimum time of occurrence in the list of all possible events. It can be proven that this minimum time follows an exponential distribution with rate parameter equal to the sum of the rate constants of all possible events. If the simulation has reached the steady state, the sum of the rate constants of all possible events will fluctuate over a constant value, and therefore the simulated time will increase linearly with the number of KMC steps.

For simulations where either of these criteria suggested that steady state might not have been reached, we conducted manual analyses and extended the simulation times as necessary (i.e., continued the simulation from the restart file).

Note S4. On the proximity factors

Although one could, in principle, determine these coefficients from first principles by running nudged-elastic-band (NEB) calculations across various surface coverages, thereby quantifying how the transition-state energy responds to shifts in initial- and final-state energies, each NEB calculation is computationally demanding. Given that our mechanism involves 80 elementary steps, systematically performing the multiple NEB calculations required to determine even a single proximity factor becomes prohibitively time-consuming.

Instead, we have adopted a commonly used simplification, assigning a proximity factor of 0.0 for non-activated reversible adsorption steps and 0.5 for all other reversible steps. To evaluate the sensitivity of our conclusions to this approximation, we focused on one of the main RDSs in our mechanism, i.e. CO_2 dissociation on HfC, and performed additional KMC simulations using both 0.5 and 0.9 for that step alone. The higher value (0.9) was chosen based on inspection of our NEB potential-energy profile for CO_2 dissociation (Figure S26A). Since the maximum of this profile lies closer to the final state (CO+O), the proximity factor for this step would be closer to 1.0 than to 0.5 in a strict Brønsted–Evans–Polanyi sense.

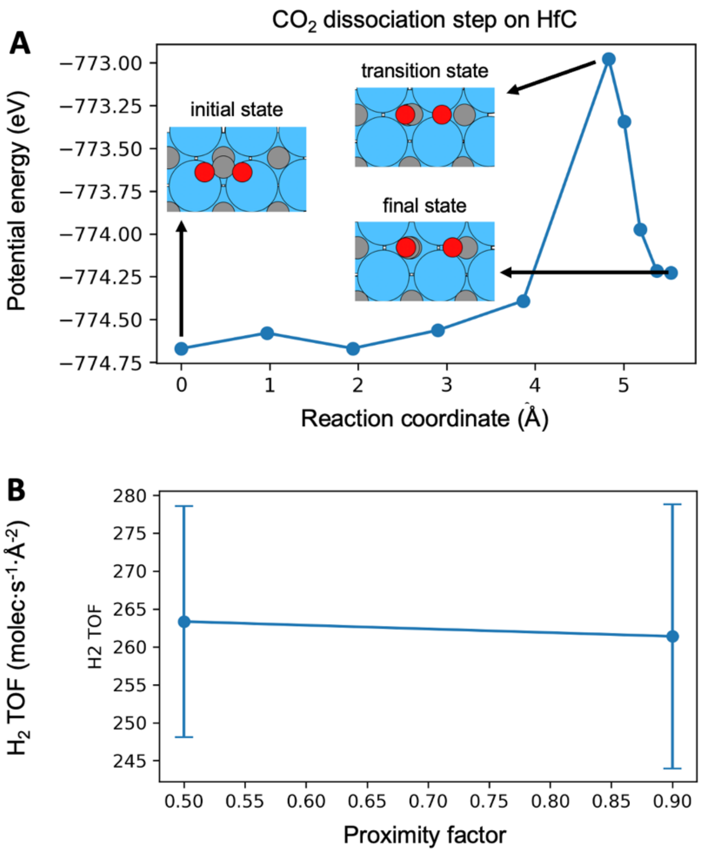

Figure S26. (**A**) Potential energy as a function of the reaction coordinate for the CO_2 dissociation step on HfC, obtained via a nudged-elastic-band (NEB) calculation. (**B**) Computed H_2 TOF as a function of the proximity factor. The error bars indicate the standard deviation from multiple KMC replicas.

After running 8 independent replicas for each proximity factor (0.5 vs. 0.9), we find no statistically significant difference in the computed TOFs. Figure S26B summarizes these results, showing that the small variations in TOF lie within the standard deviations of the simulations and thus do not alter our qualitative conclusions. Further uncertainties—from DFT functional choices to assumptions in transition-state theory—likely dominate over any minor deviations that might stem from the proximity factor alone.

Note that to get a single reaction coordinate axis from a series of NEB images we have computed the cumulative path length in configuration space from the initial image (initial state) up to each intermediate image. In other words, we computed the geometric distance each image has ‘travelled’ along the reaction pathway.

References

[1] Larsen, A. H.; Mortensen, J. J.; Blomqvist, J.; Castelli, I. E.; Christensen, R.; Dułak, M.; Friis, J.; Groves, M. N.; Hammer, B.; Hargus, C.; Hermes, E. D.; Jennings, P. C.; Jensen, P. B.; Kermode, J.; Kitchin, J. R.; Kolsbjerg, E. L.; Kubal, J.; Kaasbjerg, K.; Lysgaard, S.; Maronsson, J. B.; Maxson, T.; Olsen, T.; Pastewka, L.; Peterson, A.; Rostgaard, C.; Schiøtz, J.; Schütt, O.; Strange, M.; Thygesen, K. S.; Vegge, T.; Vilhelmsen, L.; Walter, M.; Zeng, Z.; Jacobsen, K. W. The Atomic Simulation Environment — A Python library for working with atoms. *J. Phys. Condens. Matter*. **2017**, *29*, 273002.

[2] Stamatakis, M.; Vlachos, D. G. A graph-theoretical kinetic Monte Carlo framework for on-lattice chemical kinetics. *J. Chem. Phys.* **2011**, *134*, 214115.

[3] Stamatakis, M.; Vlachos, D. G. Unraveling the complexity of catalytic reactions via Kinetic Monte Carlo simulation: Current status and frontiers *ACS Catal*. **2012** *2*, 2648-2663

[4] Danielson, T.; Sutton, J. E.; Hin, C.; Savara, A. SQERTSS: Dynamic rank-based throttling of transition probabilities in kinetic Monte Carlo simulations. *Computer Physics Communications*, **2017**, *219*, 149-163.

[5] Dybeck, E.C.; Plaisance, C. P.; Neurock, M. Generalized Temporal Acceleration Scheme for Kinetic Monte Carlo Simulations of Surface Catalytic Processes by Scaling the Rates of Fast Reactions. *Journal of Chemical Theory and Computation*, **2017**, *13*, 1525-1538.

[6] Chatterjee, A.; Voter, A. F. Accurate acceleration of kinetic Monte Carlo simulations through the modification of rate constants. *Journal of Chemical Physics*, **2010**, *132*, 194101.
